# Tunable Nanoscale Structure via Divalent Ion Identity in Charged-Neutral Polymer Blends

**DOI:** 10.1021/acsmacrolett.5c00768

**Published:** 2026-01-19

**Authors:** Hsin-Ju Wu, Aidiel Ikmal Bin Abu Hassan, Benjamin S. Bossman, Whitney S. Loo

**Affiliations:** Department of Chemical and Biological Engineering, 5228 University of Wisconsin-Madison, 1415 Engineering Drive, Madison, Wisconsin 53706, United States

## Abstract

Charged-neutral polymer blends, wherein an ion-containing polymer is blended with a neutral polymer, are potential candidates for battery electrolytes due to their improved ion transport properties and electrochemical stability. Though electrostatic interactions in charged polymer blends can theoretically stabilize ordered nanostructures analogous to those observed in neutral block copolymers, direct experimental evidence remains limited. Here, we investigate the effects of divalent cation identity on the nanoscale morphology of charged-neutral polymer blends composed of poly­(ethylene oxide) (PEO) and Mg^2+^ or Ca^2+^ ion-containing polymers, poly­[3-(methylacryloxy)­propylsulfonyl-1-(trifluoromethanesulfonylimide)] (P­(Mg­(MTFSI)_2_) or P­(Ca­(MTFSI)_2_). By tuning the size of the divalent counterion, we are able to precisely tune the ion solvation between the free cation and PEO, which acts as a solvent in this system. Differential scanning calorimetry (DSC) and small-angle X-ray scattering (SAXS) measurements reveal that Mg^2+^ and Ca^2+^ ions induce distinct structural behavior. In both systems, the blends become more miscible as the concentration of ion-containing polymer is increased indicated by increased suppression of PEO crystallinity. At the highest concentrations of P­(Mg­(MTFSI)_2_), the blends undergo microphase separation and generate nanostructures with short-ranged ordering. In contrast, calcium ions, which are more readily solvated by PEO, produce more homogeneous blends characterized by a single glass-transition temperature and featureless SAXS data. The results demonstrate the novel experimental confirmation that charged-neutral polymer blends can undergo microphase separation and show that counterion identity can be exploited as a design parameter to control nanoscale morphology.

## Graphical abstract

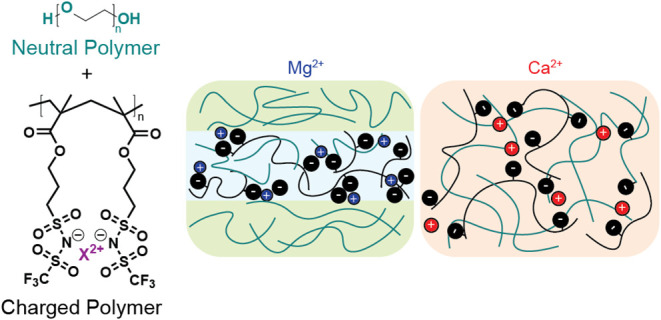

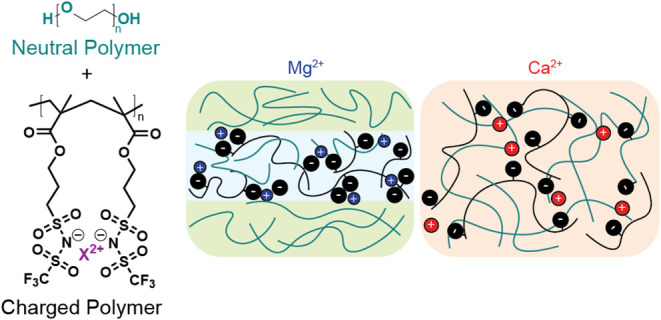

The introduction of charged species into polymer blends alters the system’s thermodynamics and enables precise control over blend nanostructure and resulting physical properties.^1–4^ While there are many classes of charged polymer blends, including blends of oppositely charged polyelectrolytes^5,6^ and neutral polymer blends doped with salt,^7–9^ the resulting thermodynamics must balance the ion solvation energy of the free ions, electrostatic interactions, and miscibility of the polymer backbones. Previous theory has predicted that charged polymer blends are capable of self-assembling into ordered phases with periodicities typically associated with block copolymers (BCPs).^10–16^ In neutral BCPs, self-assembly is primarily governed by segregation strength, χ*N*, where χ is the Flory–Huggins interaction parameter and *N* represents the degree of polymerization.^17,18^ At sufficiently high values of χ*N* for a given copolymer composition, the system will microphase separate into well-defined ordered phases. Theoretical works on charged polymer blends have maintained this framework by redefining χ to account for the energetic effects of added ions,^14^ and phase diagrams developed from such theories have predicted that charged polymer blends can form ordered phases such as body-centered cubic, hexagonally packed cylinders, and lamellae.^11,12,19^ Previous experimental studies have shown the emergence of microphase separation in blends of oppositely charged polymeric ionic liquids,^5^ polyzwitterion/polyanion blends,^20^ and polymer blends functionalized with acid/base pairs.^21^ However, the blends presented in these studies do not self-assemble into well-ordered nanostructures and instead are characterized by a single broad scattering peak corresponding to nanoscale concentration fluctuations.

One unique class of charged polymer blends is charged-neutral polymer blends, wherein an ion-containing polymer is blended with a neutral polymer, and they have recently emerged as potential candidates for battery electrolytes due to their improved ion transport properties and electrochemical stability.^22–24^ In this scheme, the competition between electrostatics and ion translational entropy can lead to either macro- or microphase separation, although experimental studies on the phase behavior of charged-neutral blends have remained limited. One prior study revealed the formation of disordered phases with low degrees of microphase separation comprised of charge-correlated domains, indicating solvation of the Li counterions by the neutral polymer.^25^ The ion translational entropy is controlled by the preference for the free counterions to be solvated by the charged or neutral polymer. In the simplest form, the ion solvation energy can be approximated by the Born energy of the ion in a given medium according to eq 1:^26^

1 $$f_{\mathrm{Born}}=\frac{q^{2}}{8\pi \epsilon \varepsilon_{0}a}$$

where ϵ is the dielectric constant of the solvating medium, ε_0_ is the vacuum permittivity, and *a* is the radius of the ion with a point charge, *q*, placed in the center. Therefore, in charged-neutral polymer blends, the free counterions have a preference to be solvated by the polymer species with the higher dielectric constant, which is typically the neutral polymer. Additionally, the Born energy shows that it is more energetically favorable to solvate ions with larger radii and lower valency. Previous studies on charged-neutral BCPs exhibit a strong dependence of phase behavior on counterion identity, wherein Li analogs were homogeneous and Mg analogs were microphase-separated.^27,28^ The authors attributed these differences to increased solvation of the monovalent Li^+^ ion, as compared to the divalent Mg^2+^ ion, by the neutral polymer block suppressing microphase separation. However, the architecture of charged-neutral BCPs changes the entropic contributions from the polymer chains compared to charged-neutral polymer blends.

In this paper, we investigate how the identity of divalent counterions tunes the nanostructures of charged-neutral polymer blends, using small-angle X-ray scattering (SAXS) and differential scanning calorimetry (DSC). The phase behavior of our charged-neutral polymer blends is dominated by ion solvation energy, where the size of the counterion enables precise tuning of the nanostructure. To the best of our knowledge, we have shown the first experimental evidence of ion-identity-induced microphase separation in charged-neutral polymer blends. The polymer blends studied consist of poly­(ethylene oxide) (PEO) and poly­[3-(methylacryloxy)­propylsulfonyl-1-(trifluoromethanesulfonylimide)] (P­(X­(MTFSI)_2_)), where X represents a Mg^2+^ or Ca^2+^ ion. The chemical structures of PEO and P­(X­(MTFSI)_2_) are shown in Figure 1. Potassium analogs of the charged monomers were synthesized via previously reported SuFEX click reactions.^29^ The charged polymers were synthesized via reversible addition–fragmentation chain-transfer (RAFT) polymerization, followed by an ion-exchange process to introduce the divalent counterions.^25^ The molecular weight of the PEO is 30 kDa (Polymer Source) and the P­(KMTFSI) is 97 kDa using end-group analysis. For each cation system, four blends were prepared with mixing ratios of 0.03 ≤ *r* ≤ 0.11, where *r* is defined as the molar ratio of counterions to ethylene oxide units, calculated as *r* = [X^2+^]/[EO] = [P­(X­(MTFSI)_2_)]/[PEO] and reflect weight fractions of P­(X­(MTFSI)_2_), *w*_X_, of 0.30 to 0.63. Additional characterization of the polymers and blends is provided in the Supporting Information.

**Figure 1:**
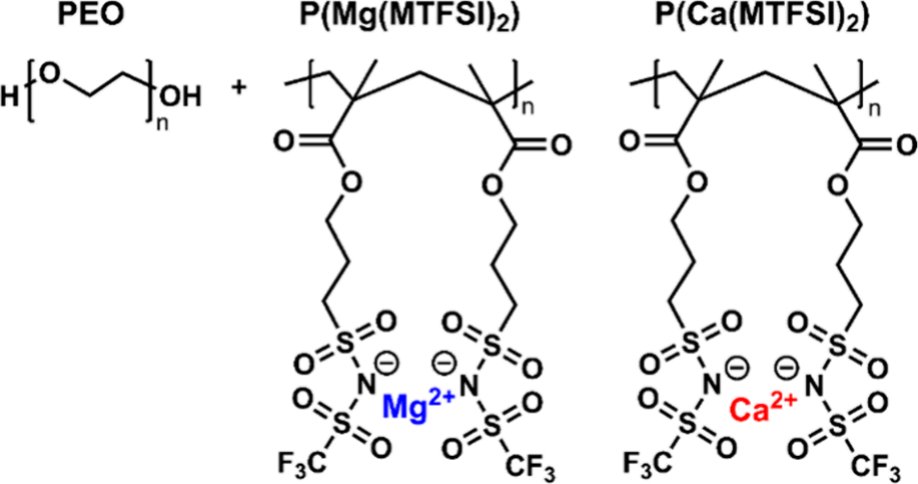
Chemical structure of poly­(ethylene oxide) (PEO), magnesium-based P­(Mg­(MTFSI)_2_), and calcium-based P­(Ca­(MTFSI)_2_).

Figure 2 presents the SAXS intensity, *I*(*q*), as a function of the scattering vector magnitude, *q*, for Mg^2+^ and Ca^2+^ blends at 90 °C, where both systems are fully amorphous. The temperature-dependent SAXS profiles for both blends are presented in Figure S11. Based on the SAXS profiles, counterion identity strongly influences nanoscale structure. The Mg^2+^ blends (Figure 2a) consistently exhibit a broad, well-defined primary scattering peak across all values of *r*, suggesting the presence of concentration fluctuations that lead to microphase separation. As *r* increases, this primary peak shifts toward lower *q*, indicating an increase in the characteristic domain spacing. For blends with *r* ≤ 0.05, one broad primary scattering peak, q*, appears in the low-*q* range. This peak becomes sharper as *r* increased to 0.08, and broad higher order peaks appear, with peak ratios of *q*/q* = √3 and √7 (Figure S13). These peaks persist at the highest concentration of charged polymer, *r* = 0.11. However, these peaks are too broad to index properly and identify a specific nanostructure. This observation demonstrates that charged-neutral polymer blends can undergo microphase separation and self-assemble into nanostructures at length scales typically observed in BCPs.

**Figure 2:**
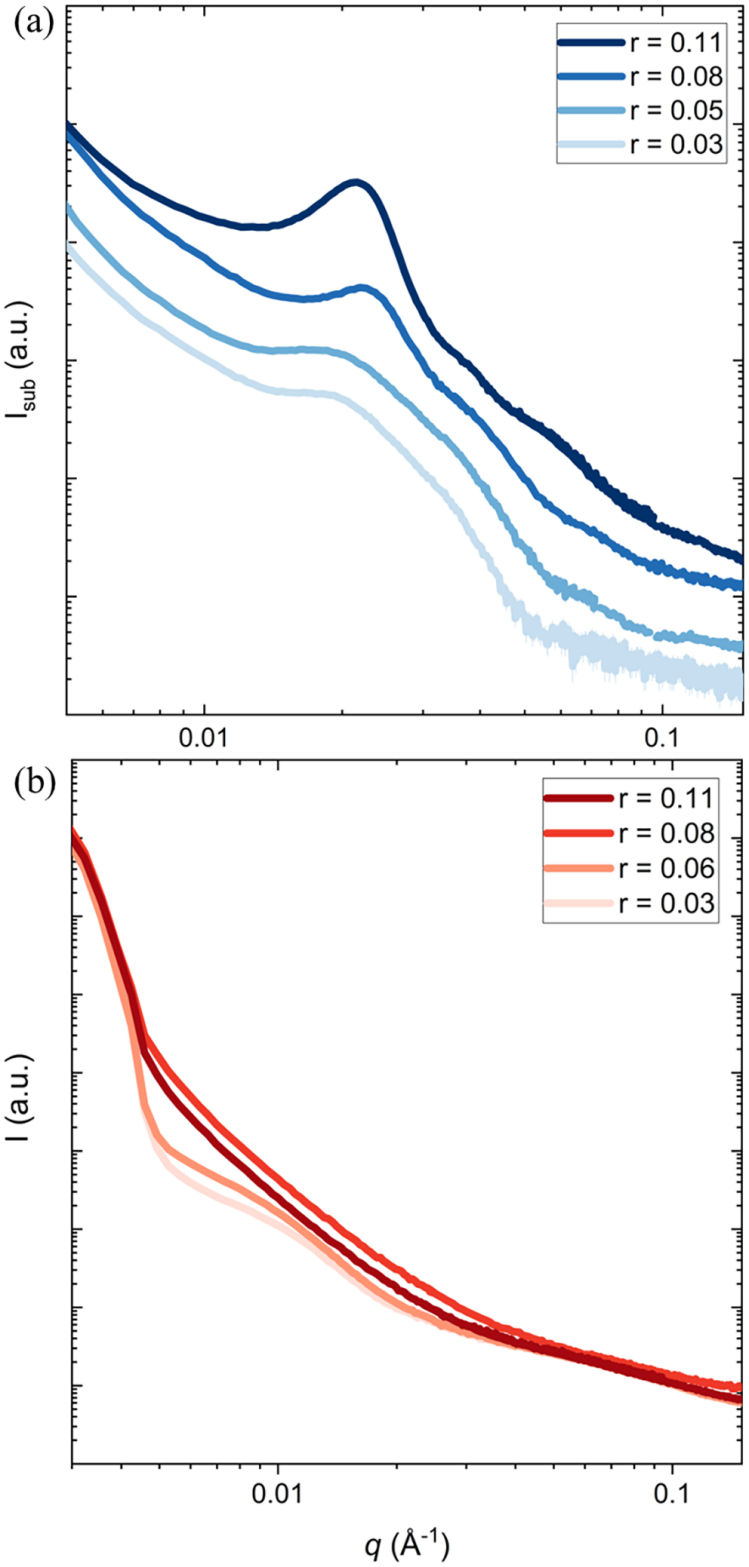
Small-angle X-ray scattering (SAXS) profiles of PEO blends with (a) P­(Mg­(MTFSI)_2_) and (b) P­(Ca­(MTFSI)_2_) as a function of the mixing ratio, *r*, at 90 °C. Both blends are amorphous at this temperature. The data in (a) were corrected by subtracting the scattering contribution from the Kapton tape, whereas those in (b) are shown as measured without subtraction. Error bars represent standard deviation and are smaller than the data points.

In contrast, the Ca^2+^ blends (Figure 2b) exhibit a weak, broad peak at low values of *r* (0.03 and 0.06), which disappears as *r* increases to 0.08 and 0.11. The broad scattering peaks are similar in intensity to ionomer peaks observed in other charged polymer systems, but appear at much lower values of *q*, corresponding to charge correlations with large length-scales.^27,30–33^ The absence of a distinct scattering peak indicates that the Ca-based systems are fully homogeneous at high P­(Ca­(MTFSI)_2_) concentrations. The effect of mixing ratio on scattering is dependent on counterion identity. In the Mg^2+^ case, as *r* increases, corresponding to an increase in the concentration of charged polymer, the scattering intensity becomes stronger and the peaks become sharper, whereas in the Ca^2+^ case, as *r* increases, the scattering intensity decreases. Notably, the scattering profiles of microphase-separated Mg^2+^ blends differ qualitatively from those previously reported for Li-analogs of charged-neutral polymer blends,^25^ which exhibit only a weak low-*q* shoulder that disappears at higher *r*, which is in qualitative agreement with the Ca^2+^ system. Overall, the SAXS results highlight the critical role of counterion identity in directing the morphology, due to the balance between cation solvation by PEO and cation dissociation with TFSI^–^ anions.

To quantitatively assess the morphology of the microphase separated P­(Mg­(MTFSI)_2_)/PEO blends, the SAXS data were analyzed using the Teubner–Strey (T-S) model (eq 2), which was originally developed for oil/water/surfactant microemulsions^34^ as given by

2 $$I\left(q\right)=\frac{1}{a+bq^{2}+cq^{4}}$$

The T-S model has been previously applied to characterize nanostructures of polymer systems such as a mixture of two incompatible homopolymers and a diblock copolymer,^35,36^ Mg-containing single-ion conducting block copolymer,^28^ and polyelectrolyte complexes.^3^ The fits to the scattering profiles are shown in Figure S12. The obtained fitting parameters (*a*, *b*, and *c*) were subsequently used to extract two characteristic length scales that describe the nanoscale morphology of the blends, the domain spacing, *d*, and correlation length, ξ, as well as the amphiphilicity factor, *f*_a_, which characterizes the stability of the microemulsion (eqs 4–6).

3 $$d=2\pi {\left[\frac{1}{2}{\left(\frac{a}{c}\right)}^{1/2}-\frac{b}{4c}\right]}^{-1/2}$$

4 $$\xi ={\left[\frac{1}{2}{\left(\frac{a}{c}\right)}^{1/2}+\frac{b}{4c}\right]}^{-1/2}$$

5 $$f_{a}=\frac{b}{\sqrt{4ac}}$$

Figure 3a shows the domain spacing, *d*, and correlation length, ξ, obtained from fitting the T-S model as a function of *w*_*Mg*_ at 90 °C. The domain spacing characterizes the length scale of phase separation in the system, while the correlation length reflects the spatial distance over which composition fluctuations remain correlated, beyond which the structural correlations decay exponentially.^37,38^ The values of *d* fitted with the T-S model are quantitatively similar to those calculated directly from the scattering profile according to *d*_0_ = 2π/q* (Table S3). As *r* increases, corresponding to an increase in *w*_Mg_, *d* generally decreases from 37.3 to 29.8 nm, indicating that the length-scale of phase separation decreases. Fitting results for other temperatures are provided in the Supporting Information (Table S3 and Figure S14), and generally, *d* increases with increasing temperature, corresponding to an expansion of the phases. Conversely, ξ increases with increasing *w*_Mg_ from 8.3 to 26.6 nm, indicating that the overall size of the charge correlated regions increases as the concentration of charged polymer is increased. Figure 3b shows the effect of mixing ratio on the amphiphilicity factor, *f*_a_, which can be used as an indicator of the stability of microemulsion-like systems.^3,39^ When *f*_a_ < −1, the amphiphilicity becomes so strong that the microemulsion phase is destabilized and generate a lamellar-like phase.^40^ When −1 < *f*_a_ < 0, the system forms a “good” microemulsion, characterized by weaker surface tension, which leads to a bicontinuous structure. As *w*_Mg_ increases, *f*_a_ decreases from −0.32 to −0.94, indicating an increase in ordering with an increasing concentration of charged polymer. The structure of the blends, characterized by *f*_a_, is independent of temperature at *r* ≥ 0.05, indicating that the nanostructures formed are highly stable. The SAXS profiles for the *r* = 0.03 and *r* = 0.06 Ca-analogs were also fit to eq 2, and the results are provided in the Supporting Information (Figures S15 and S16). Overall, both *d* and ξ are larger and more temperature-sensitive than what is observed for the Mg-analogs, indicating formation of a less-ordered nanostructure.

**Figure 3:**
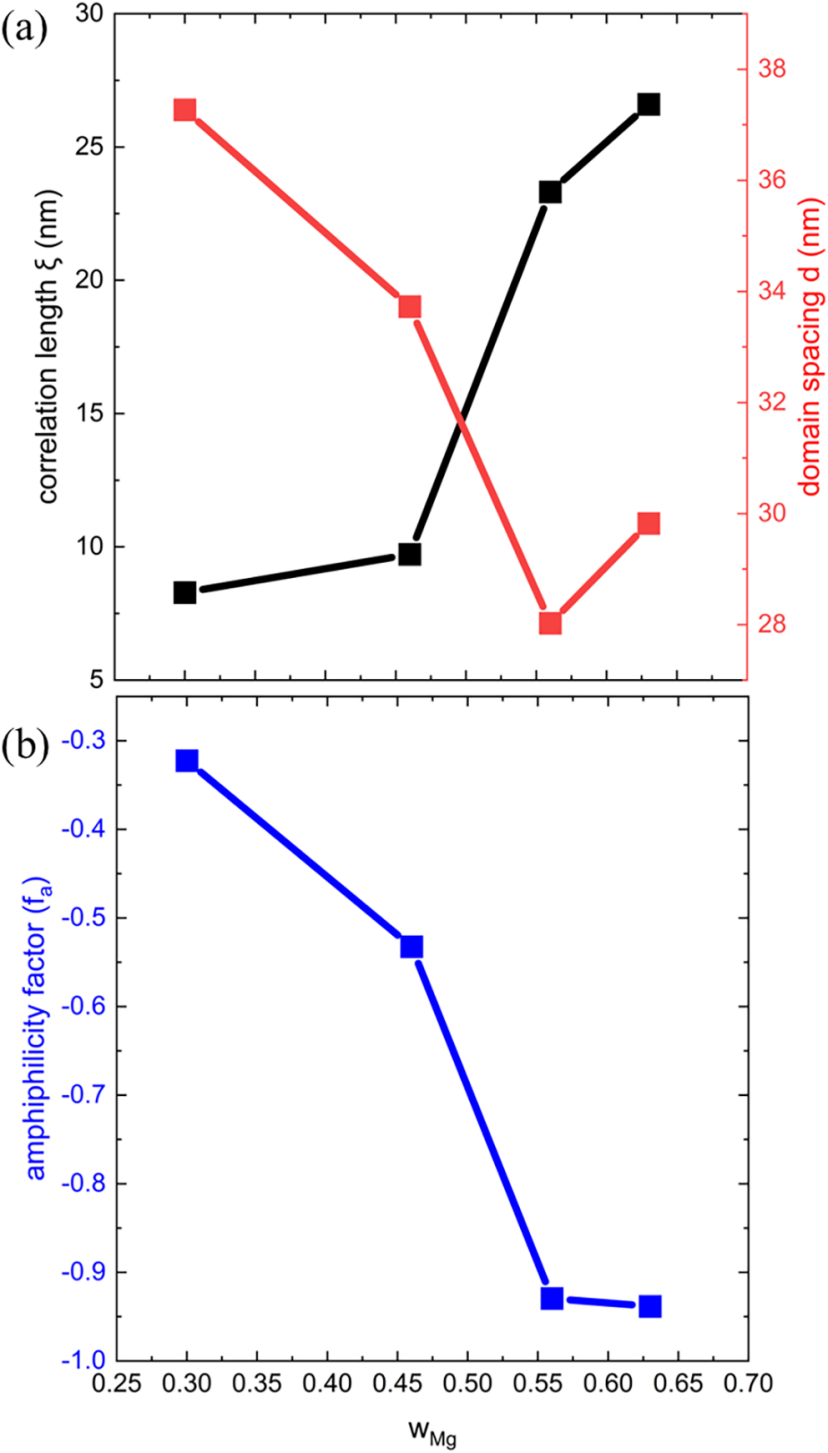
(a) Domain size (*d*, red), correlation length (ξ, black), and (b) amphiphilicity factor (*f*_a_, blue) of PEO/P­(Mg­(MTFSI)_2_) obtained from fitting their scattering traces to the Teubner–Strey (T-S) equation at 90 °C.

Based on the observed dependence of *d*, ξ, and *f*_a_ on *r*, we propose possible pathways for structural evolution in this system. The scattering profiles were analyzed using the T-S model, originally developed for oil/water/surfactant microemulsions. By analogy, PEO can be considered as the “oil”, the charged polymer as the “water”, and the counterions as the “surfactants” that improve mixing through favorable ion-dipole and electrostatic interactions. At *r* = 0.03, where PEO is the majority component, the system exhibits microemulsion-like structures. The strong electrostatic interaction between Mg^2+^ and anions on the charged polymer prevents complete cation solvation by PEO; however, ion-dipole interactions still maintain a close association between PEO and Mg^2+^, stabilizing the microemulsion. As *r* increases, the fraction of charged polymer rises, and the PEO layer becomes thinner, leading to a reduction in *d* and a transition toward a more lamellar organization, with *f*_a_ approaching −1. At high values of *r*, the microphase separation becomes more pronounced. Additional incorporation and entanglement of charged polymer chains also increase ξ to match the value of *d*, consistent with the formation of lamellar-like structures. Although we cannot confirm the precise morphology from the SAXS profiles, the presence of higher order scattering peaks, which have not been previously observed in charged polymer blends, and the results from fitting the T-S model indicate that our system undergoes microphase separation into nanostructures with short-range ordering when *r* ≥ 0.08. Overall, these observations reveal the coupled influence of electrostatic interactions and blend composition on governing the structure and stability of phase-separated morphologies in charged-neutral polymer blends.

Figure 4 presents the DSC thermograms of PEO blended with (a) P­(Mg­(MTFSI)_2_) and (b) P­(Ca­(MTFSI)_2_) at various mixing ratios, *r*. DSC analysis is commonly used to assess polymer blend miscibility and, here, to evaluate differences in thermal phase behavior induced by the divalent counterions. The DSC data for the homopolymer components are provided in Figure S7. For the Mg-based blends (Figure 4a), a distinct PEO melting endotherm near 64 °C is observed across all values of *r*, indicating phase separation into a mostly pure, crystalline PEO domain and an amorphous PEO/P­(Mg­(MTFSI)_2_) complex phase. We hypothesize that this phase separation is on the nanoscale, as we do not observe the *T*_g_ value corresponding to pure P­(Mg­(MTFSI)_2_), at 86 °C, in the DSC traces for the blends. Therefore, we classify these systems as macroscopically miscible. In contrast, the thermal properties of the Ca-based blends (Figure 4b) are strongly dependent on *r*. While the PEO crystalline peak is evident at a low content of charged polymer (*r* = 0.03 and 0.06), it is completely suppressed at the higher mixing ratios (*r* = 0.08 and 0.11). At these elevated calcium loadings, the thermograms instead show a single well-defined *T*_g_, confirming the transition to a fully amorphous state. In both sets of blends, the strength of the melting transition changes with *r*, and the fractional PEO crystallinity in PEO/P­(X­(MTFSI)_2_), *X*_c_, can be calculated according to eq 6:

6 $$X_{c}=\frac{\Delta H_{m,\mathrm{blend}}}{w_{\mathrm{PEO}}\Delta H_{m,\mathrm{PEO}}^{0}}$$

where Δ*H*_m,blend_ is the experimental melting enthalpy of the blend, *w*_PEO_ is the weight fraction of PEO within the blend, and Δ*H*_m,PEO_^0^ is the ideal melting enthalpy of a fully crystalline PEO taken as 206 J/g in this study.^41,42^ Figure 4c plots *X*_c_ as a function of *w*_*x*_ for the Mg-analogs (blue) and Ca-analogs (red). For both systems, *X*_c_ decreases with increasing *w*_*x*_, indicating that as the concentration of charged polymer increases, the degree of PEO crystallinity decreases due to increased solvation of the cations into the PEO domains. Therefore, as *r* increases, the degree of mixing between PEO and P­(X­(MTFSI)_2_) also increases, which matches previous studies of Li-containing charged-neutral polymer blends.^25^ Additionally, we observe that the Ca^2+^ ions more effectively mix with PEO due to its consistently lower values of *X*_*c*_, which is in agreement with the SAXS results.

**Figure 4:**
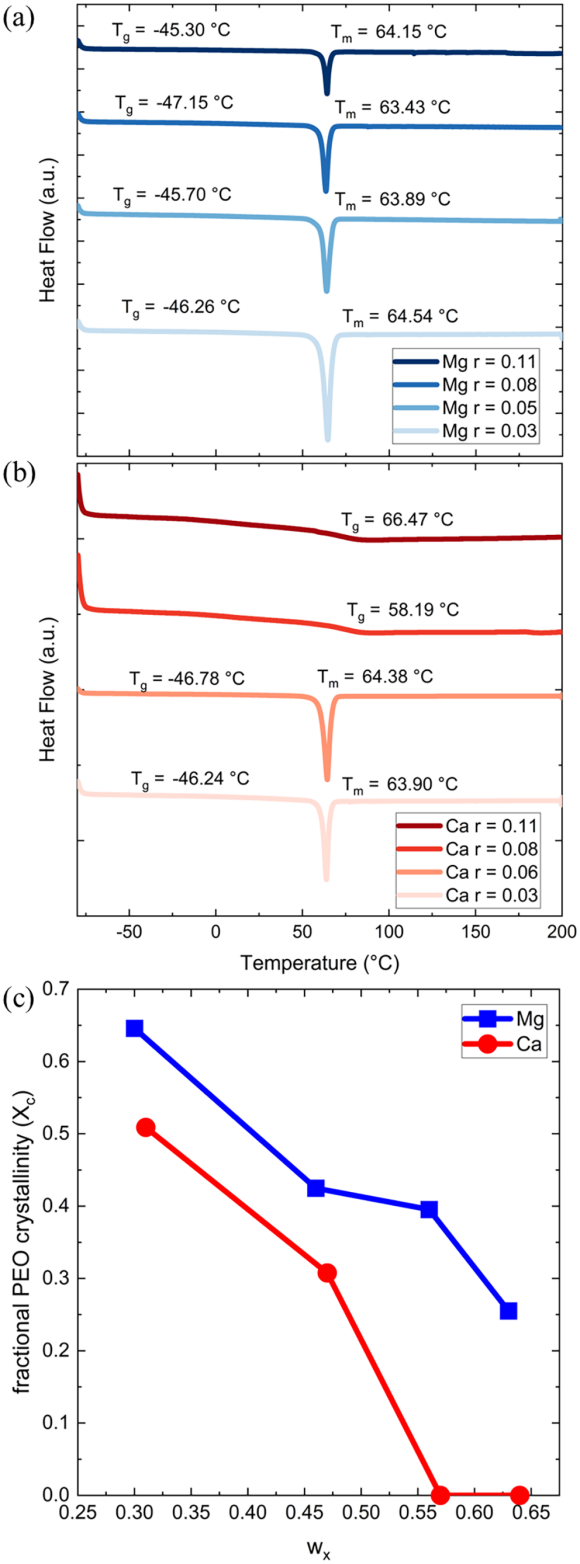
Differential scanning calorimetry thermograms of PEO with (a) P­(Mg­(MTFSI)_2_) and (b) P­(Ca­(MTFSI)_2_) at various mixing ratios, *r*. (c) Fractional PEO crystallinity, *X*_c_, in PEO/P­(Mg­(MTFSI)_2_) and PEO/P­(Ca­(MTFSI)_2_) versus *w*_*x*_.

Based on the DSC and SAXS results, we conclude that as *r* increases, corresponding to an increase in charged polymer, mixing between the PEO and P­(X­(MTFSI)_2_) chains becomes more energetically favorable. However, the resulting nanoscale structures of the charged-neutral blends are highly dependent on counterion identity. Figure 5 shows a schematic illustration of the proposed nanostructures of PEO/P­(X­(MTFSI)_2_) blends at *r* = 0.11 with distinct divalent cations. We hypothesize that the changes in nanostructure are due to the differences in cation size, which affect both the ion solvation energy and electrostatic interactions. The larger Ca^2+^ ion shows weaker electrostatic binding and enhanced ion dissociation as compared to the smaller Mg^2+^ ion, resulting in a more homogeneous morphology at low ion concentration. This is determined by the suppression of the PEO crystallization at moderate values of *r* as well as the featureless SAXS patterns indicative of a homogeneous morphology. As *r* increases, both the DSC and SAXS data indicate increased mixing between the PEO and the P­(Ca­(MTFSI)_2_) chains. Interpretation of the data for the Mg^2+^ case is more complicated. The DSC data show a suppression of PEO crystallinity with increasing *r*, however, the scattering intensity increases with increasing *r*. Upon quantitative analysis of the scattering data, we find that the Mg^2+^ blends undergo a transition from a disorganized microemulsion-like state to a more correlated lamellar-like state. Therefore, although the degree of microphase separation increases with increasing *r*, the degree of mixing between PEO and P­(Mg­(MTFSI)_2_) chains also increases as evident by the decrease in domain size with increasing *r*. While the resulting nanoscale structure of charged-neutral blends is highly dependent on counterion identity, all blends exhibit increases in mixing between the two polymer components as the concentration of charged polymer increases. These experimental results also agree with the theoretical predictions. Phase diagrams for charged-neutral polymer blends developed by Grzetic were asymmetric with respect to the blend composition.^13^ The thermodynamic boundary to undergo microphase separation appeared at lower values of χ*N* when *w*_*x*_ > 0.5. Our experimental results on the Mg-analogs agree with this theory where we see the formation of ordered phases at high values of *r* while the nanoscale structure is highly disordered at low values of *r*.

**Figure 5:**
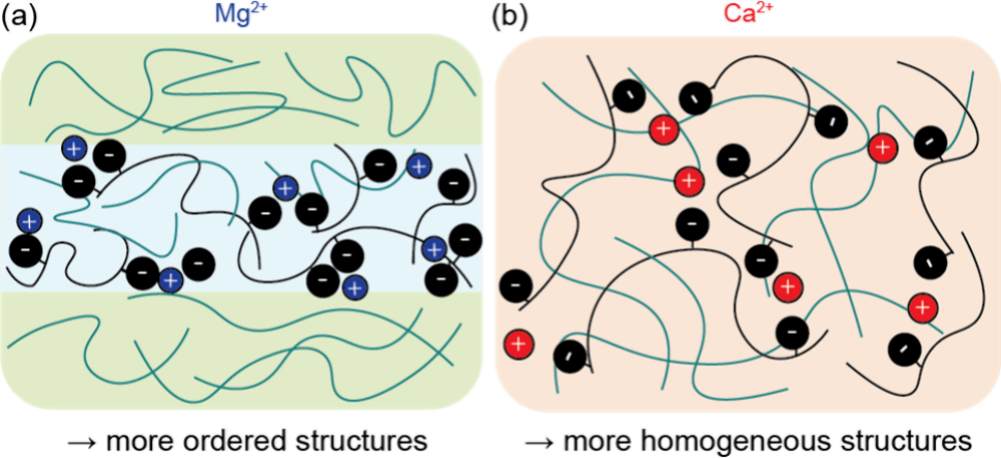
Schematic illustration of microstructures in single-ion conducting polymer blends with (a) P­(Mg­(MTFSI)_2_) and (b) P­(Ca­(MTFSI)_2_).

In summary, we have demonstrated that the ion dissociation strength strongly influences the nanoscale structures and report the first experimental evidence for electrostatically stabilized microphases in charged-neutral blends. Mg^2+^ ions, with their smaller ionic radius and stronger electrostatic interactions, induce short-range ordering, indicated by SAXS and further supported by the sharp PEO melting peaks observed in DSC. In contrast, Ca^2+^ ions exhibit weaker electrostatic interactions and increased solubility in PEO, leading to more homogeneous and miscible blends, similar to the observations in Li^+^ systems. These findings also highlight that charged polymer blends can achieve structural features typically observed in BCPs by appropriately selecting the counterion as well as its concentration and utilizing their dissociation characteristics.

## Supplementary Material



## Notes

Materials and Methods, NMR, ICP-OES, DSC, and related scattering details (PDF)
