# Supplementary material for: Tunable Nanoscale Structure via Divalent Ion Identity in Charged-Neutral Polymer Blends
Source: ACS Macro Lett. 2026 Jan 19;15(2):288–94. doi: 10.1021/acsmacrolett.5c00768 (PMC12918717; doi:10.1021/acsmacrolett.5c00768)
Supplement: Supplementary file 1 [file mz5c00768_si_001.pdf]

Supporting Information for:

Tunable Nanoscale Structure via Divalent Ion Identity in Charged-Neutral Polymer Blends

*Hsin-Ju Wu, Aidiel Ikmal Bin Abu Hassan, Benjamin S. Bossman, Whitney S. Loo**

Department of Chemical and Biological Engineering, University of Wisconsin—Madison, 1415 Engineering Drive, Madison, Wisconsin 53706, United States

*Email: wloo@wisc.edu

Materials and Methods

Materials

Potassium 3-sulfopropyl methacrylate (98%, Sigma-Aldrich, USA), oxalyl chloride (98%, Sigma Sigma-Aldrich, USA), potassium bifluoride (KHF_2, 99%, Sigma-Aldrich, USA), trifluoromethanesulfonamide (95%, Sigma Sigma-Aldrich, USA), acetonitrile (ACN, ≥99.5%, Sigma-Aldrich, USA), dimethylformamide (DMF, anhydrous, 99.8%, Sigma-Aldrich, USA), dichloromethane (DCM, anhydrous, ≥99.8%, contains 40~150 ppm amylene as stabilizer, Sigma-Aldrich, USA), diethyl ether (Fisher Scientific, USA), 2-cyano-2-propyl benzodithioate (>97%, Sigma-Aldrich, USA), acetone (≥99.5%, Sigma-Aldrich, USA), dimethyl sulfoxide-d_6 (DMSO-d_6, 99.9 atom % D, contains 0.03% (v/v), Sigma-Aldrich, USA), sodium sulfate (Na_2SO_4, ≥99.0%, anhydrous, Sigma-Aldrich, USA), calcium carbonate ($CaCO_3$, ≥99.0%, Sigma-Aldrich, USA), butylated hydroxytoluene (BHT, ≥99%, Sigma-Aldrich, USA), potassium carbonate (K_2CO_3, ≥ 99.0%, Sigma-Aldrich, USA), calcium gluconate (monohydrate, DOT Scientific, USA), methanol (≥99.8%, Sigma-Aldrich, USA), magnesium chloride ($MgCl_2$, 99%, Sigma-Aldrich, USA), calcium chloride ($CaCl_2$, 99%, Sigma-Aldrich, USA), and sodium chloride (NaCl, ≥99%, Sigma-Aldrich, USA) were used as received. Azobisisobutyronitrile (AIBN, Sigma-Aldrich, USA) was purified by recrystallization from methanol, involving dissolution at 50 °C followed by cooling in a freezer to induce crystal formation. Poly(ethylene oxide) (PEO, Polymer Source, Canada) was dried under vacuum in the antechamber of a glovebox for 24 h and then transferred into the glovebox.

Synthesis of P(Mg(MTFSI)$_2$) and P(Ca(MTFSI)$_2$)

The potassium sulfonyl(trifluoromethanesulfonyl)imide methacrylate (KMTFSI) monomer was synthesized following the procedure previously reported.[1] The poly[potassium sulfonyl(trifluoromethanesulfonyl)imide methacrylate], P(KMTFSI), was synthesized via

reversible addition-fragmentation chain-transfer (RAFT) polymerization. Briefly, 7.5 g of KMTFSI, 0.006 g of azobisisobutyronitrile, and 0.08 g of 2-cyano-2-propyl benzodithioate were fully dissolved in 25 mL of dimethylformamide (DMF) in a 100 mL Schlenk flask. After three cycles of freeze-pump-thaw, the flask was filled with nitrogen and heated at 70°C for 24 hr. The product was precipitated by diethyl ether and recovered with methanol three times. The polymer was dried at 60°C under high vacuum for 24 hr. P(KMTFSI) and 4 equivalents of 0.2 M alkaline metal chlorides (Mg^{2+}, Ca^{2+}) solutions were stirred at room temperature overnight to facilitate ion exchange of potassium ions with lithium ions. The excess XCl_2 (X = Mg^{2+}, Ca^{2+}) was removed by dialysis against deionized water (MWCO: 1 kDa) for one week. The resulting P(XMTFSI) powders were obtained after freeze-drying for 4 days and subsequently stored in an argon glovebox (MBraun) with water and oxygen levels maintained at less than 1 ppm.

Nuclear Magnetic Resonance (NMR)

^{1}H- and ^{19}F-NMR spectra were recorded using a Bruker AVANCE III 400 NMR spectrometer.

Polymer Blend Preparation

The polymer blends investigated in this study consist of poly(ethylene oxide) (PEO) and $P(X(MTFSI)_2)$ (X = Mg^{2+} or Ca^{2+}). A series of PEO/$P(X(MTFSI)_2)$ blends with varying mixing ratios were prepared inside an argon-filled glovebox, with their compositions summarized in **Table S2**. PEO and $P(X(MTFSI)_2)$ were each dissolved separately in anhydrous methanol at a concentration of 10 mg/mL and stirred overnight. The blend composition is defined by $r = [X^{2+}]/[EO] = [\mathrm{P(X(MTFSI)_2)}]/[PEO]$, representing the ratio of divalent cations to ethylene oxide units. The two solutions were combined and stirred at 70 °C for at least 12 hours to ensure homogeneity. After thorough mixing, the vial caps were removed, and the solvent was allowed to evaporate slowly on a hot plate at 70 °C. The resulting polymer films were further dried in a vacuum oven at 90 °C for 72 h to remove the residual solvent.

Inductively Coupled Plasma Optical Emission Spectroscopy (ICP-OES)

The ion content in $P(Mg(MTFSI)_2)$ and $P(Ca(MTFSI)_2)$ was quantified by ICP-OES analysis. Samples were prepared by dissolving polymers in deionized water at five concentrations (15.625, 31.25, 62.5,125, and 250 ppm) in conical tubes. ICP-OES measurements were done using Agilent 5800 ICP-OES system. Each sample was analyzed in triplicate under the following conditions: axial viewing mode, 5 second read time, and Nebulizer flow rate of 0.7 L/min. Ion concentrations were determined using the following emission wavelengths: 317.93 nm for Ca^{2+} ions, 766.49 nm for K^+ ions, and 279.80 nm for Mg^{2+} ions. Before sample measurements, calibration standards were prepared using KCl, $MgCl_2$, and $CaCl_2$ at concentrations ranging from 10 ppm to 300 ppm. These calibration standards were used to generate calibration curves from which ion concentrations were determined.

Differential Scanning Calorimetry (DSC)

Polymer samples ranging from 2 to 10 mg were hermetically sealed in aluminum pans in a glovebox. DSC experiments were performed using a TA Instruments Q100 instrument using two heating and cooling cycles. The heating rate was 10 °C/min and the cooling rate was 5 °C/min over

a temperature range of -80 °C to 200 °C. The melting temperature (T_m) and T_g were determined from the second heating cycle.

X-ray Scattering

Samples were prepared by melting the polymer blends at 90 °C into a stainless-steel holder with a 4 mm inner diameter and a 0.554 mm wall thickness. The samples were annealed at 90 °C in a vacuum oven for at least 24 h to remove bubbles. After this, the heater was turned off and the samples were slowly cooled under vacuum. Kapton polyimide tape was used to seal the prepared samples. Temperature-dependent measurements were performed using a Xeuss 3.0 system. Measurements were performed every 20 °C from 50 °C to 130 °C with an exposure time of 1200 seconds in the standard mode. Samples were allowed to equilibrate at each temperature for 20 minutes prior to measurement. The resulting 2D scattering patterns were isotropic and were azimuthally integrated into 1D profiles using XSACT Pro advanced data analysis software. Background intensities from the Kapton tape were subtracted from the total intensity. The intensity, $I(q)$, was recorded as a function of the magnitude of the scattering wavevector, defined as $q = \frac{4\pi}{\lambda}\sin(\frac{\theta}{2})$, where θ is the scattering angle and λ is the X-ray wavelength.

Figures and Tables

Table S1. The library of P(X(MTFSI)$_2$)/PEO blends where X is either Mg or Ca. w_{ion} is the weight fraction of charged polymer.

Sample	r	w_{ion}
PEO/P(Mg(MTFSI)$_2$) (0.03)	0.03	0.30
PEO/P(Mg(MTFSI)$_2$) (0.05)	0.05	0.46
PEO/P(Mg(MTFSI)$_2$) (0.08)	0.08	0.56
PEO/P(Mg(MTFSI)$_2$) (0.11)	0.11	0.63
PEO/P(Ca(MTFSI)$_2$) (0.03)	0.03	0.31
PEO/P(Ca(MTFSI)$_2$) (0.06)	0.06	0.47
PEO/P(Ca(MTFSI)$_2$) (0.08)	0.08	0.57
PEO/P(Ca(MTFSI)$_2$) (0.11)	0.11	0.64

Table S2. Peak locations in $I_{sub} \times q^2$ versus q profiles of PEO/P(Mg(MTFSI)$_2$) blends.

r	T (°C)	1st peak ($Å^{-1}$)	2nd peak ($Å^{-1}$)	3rd peak ($Å^{-1}$)	1st peak ratio	2nd peak ratio	3rd peak ratio
0.03	90	0.02055	-	-	1	-	-
	110	0.01987	-	-	1	-	-
	130	0.01749	-	-	1	-	-
0.05	70	0.02224	-	-	1	-	-
	90	0.021224	-	-	1	-	-
	110	0.01987	-	-	1	-	-
	130	0.01885	-	-	1	-	-
0.08	70	0.024959	0.034807	0.042278	1	1.39457	1.69390
	90	0.0236	0.03922	-	1	1.66186	-
	110	0.02088	0.034128	-	1	1.63448	-
	130	0.01919	-	-	1	-	-
0.11	70	0.02564	0.04466	-	1	1.74181	-
	90	0.022224	0.03752	0.05725	1	1.68826	2.57604
	110	0.02021	0.03583	0.05247	1	1.77288	2.59624
	130	0.01817	0.04567	-	1	2.51348	-

Table S3. Teubner-Strey (T-S) fitting parameters for PEO/P(Mg(MTFSI)$_2$) blends.

r	T (°C)	a	b ($Å^2$)	c ($Å^4$)	d (Å)	ξ (Å)	f_a	$d_0 = \frac{2\pi}{q_0}$ (Å)
0.03	90	1.649 ± 1.204	-2473.4 ± 3910.5	8922841 ± 8434327	372.7	82.9	-0.32	326.8
	110	2.927 ± 0.131	-9264.6 ± 553.1	18818300 ± 653590	351.1	116.2	-0.62	329.2

	130	1.949 ± 0.184	-7691.2 ± 992.6	21606400 ± 1545218	406.3	127.8	-0.59	384.8
0.05	70	3.203 ± 0.274	-7768.2 ± 1012.5	14232900 ± 1152764	325.1	99.6	-0.58	288.9
	90	1.096 ± 0.019	-2577.2 ± 88.6	5344053 ± 90369	337.3	97.2	-0.53	380.6
	110	0.893 ± 0.053	-2510.8 ± 229.5	5942161 ± 291429	363.1	106.5	-0.54	347.2
	130	0.726 ± 0.067	-2367.2 ± 321.9	6183975 ± 442661	384.6	115.0	-0.56	380.6
0.08	70	3.913 ± 0.644	-12266.1 ± 2264.2	12339300 ± 2011635	272.9	173.9	-0.88	265.0
	90	4.278 ± 0.519	-15259.5 ± 2036.3	15754900 ± 1923310	280.2	233.2	-0.93	279.6
	110	2.127 ± 0.111	-8993.1 ± 531.5	12016900 ± 634597	315.2	207.4	-0.89	314.6
	130	1.107 ± 0.068	-5198.7 ± 375.2	8987235 ± 536110	351.2	180.0	-0.82	343.6
0.11	70	1.929 ± 0.113	-5779.6 ± 373.8	4809912 ± 284707	253.0	248.4	-0.95	252.0
	90	1.000 ± 0.060	-4096.8 ± 271.3	4766786 ± 285726	298.2	266.0	-0.94	296.5
	110	0.781 ± 0.039	-3791.2 ± 207.4	5194121 ± 257002	323.9	295.5	-0.94	322.7
	130	0.409 ± 0.011	-2455.0 ± 73.3	4199552 ± 112716	361.4	316.9	-0.94	361.9

Table S4. Teubner-Strey (T-S) fitting parameters for PEO/P(Ca(MTFSI)$_2$) blends.

r	T (°C)	a	b (Å^2)	c (Å^4)	d (Å)	ξ (Å)	f_a
	70	0.426 ± 0.084	-1725.0 ± 1389.9	63558800 ± 89256	909.5	171.1	-0.17
0.03	90	0.368 ± 0.106	-3342.1 ± 2494.9	109644000 ± 20274300	1038.5	216.4	-0.26
	110	0.066 ± 0.017	-155.0 ± 635.2	91192900 ± 5092631	1684.1	276.6	-0.03
	130	0.080 ± 0.016	-3415.7 ± 740.6	156275000 ± 7715188	1534.6	414.0	-0.48
0.05	70	0.040 ± 0.004	924.0 ± 107.0	29643800 ± 581048	1942.8	196.0	0.43
	90	0.091 ± 0.009	-484.2 ± 252.7	59294000 ± 1538082	1349.6	238.4	-0.10
	110	0.084 ± 0.012	-1958.1 ± 421.7	107225000 ± 3312354	1458.1	325.5	-0.33
	130	0.161 ± 0.045	-8976.5 ± 2328.1	269965000 ± 35574900	1387.2	507.0	-0.68

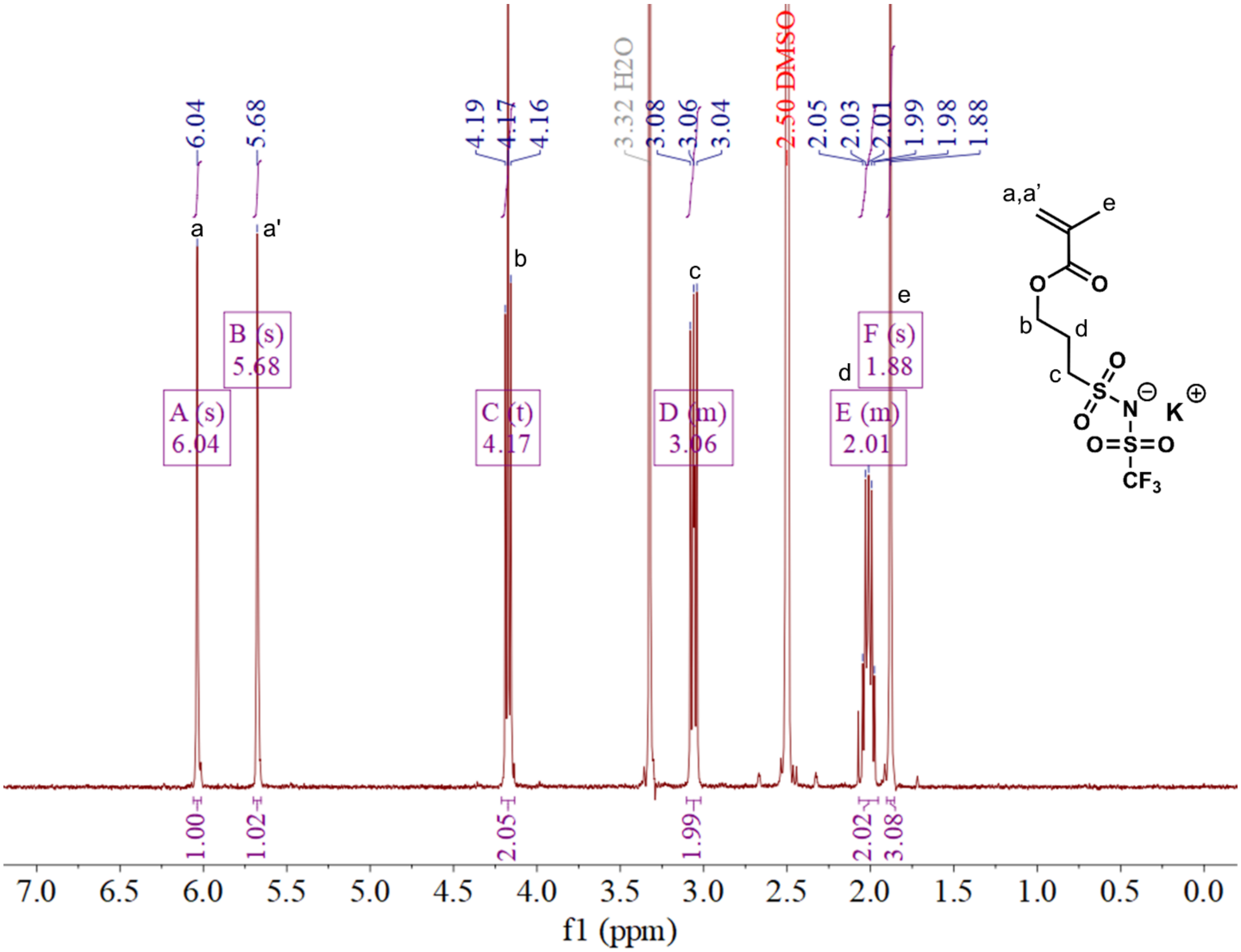

Figure S1. ^{1}H-NMR spectrum of KMTFSI (400 MHz, DMSO): δ 6.04 (s, 1H), 5.68 (s, 1H), 4.17 (t, J = 6.4 Hz, 2H), 3.10 – 3.02 (m, 2H), 2.07 – 1.95 (m, 2H), 1.88 (s, 3H).

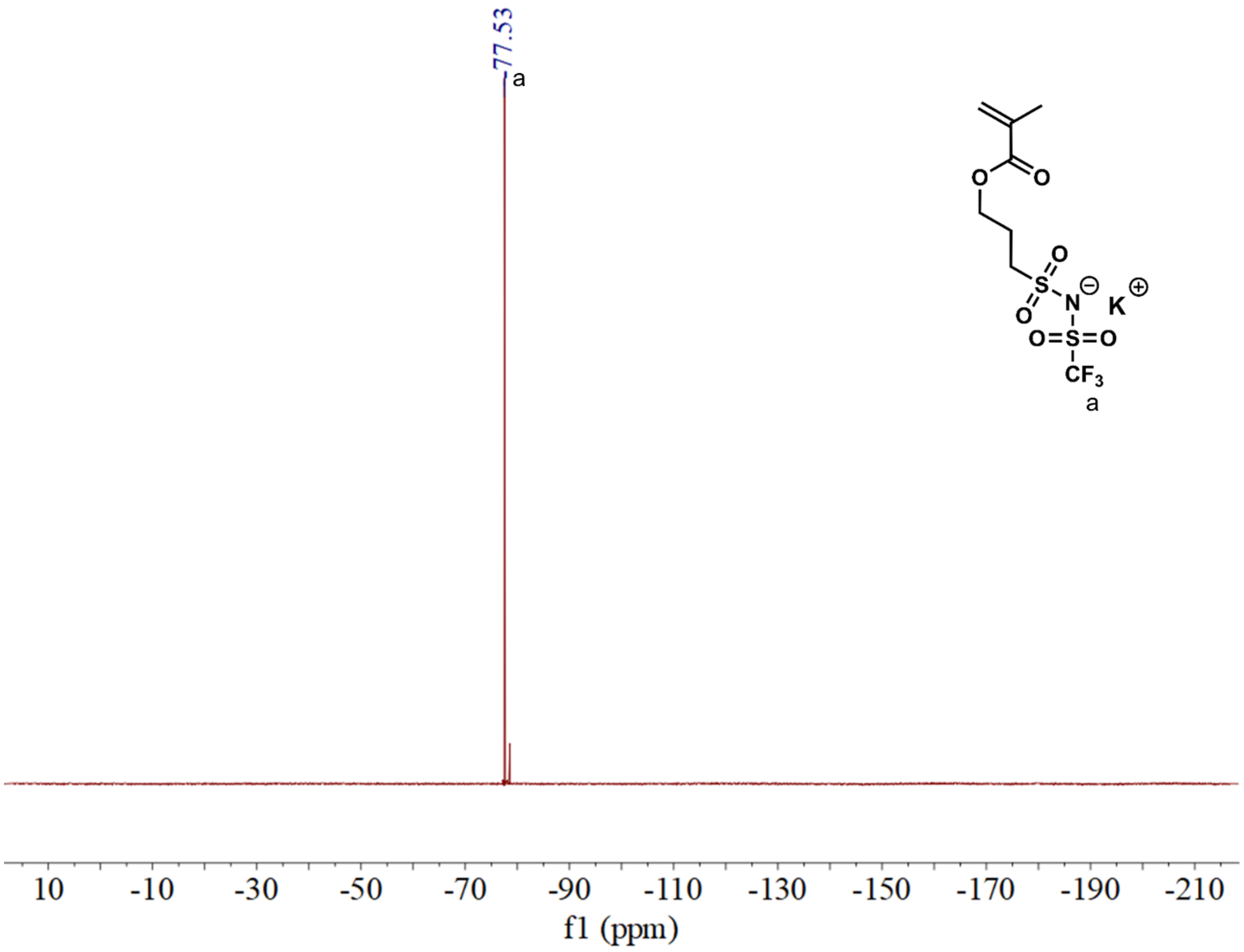

Figure S2. ^{19}F-NMR spectrum of KMTFSI (377 MHz, DMSO): δ -77.53.

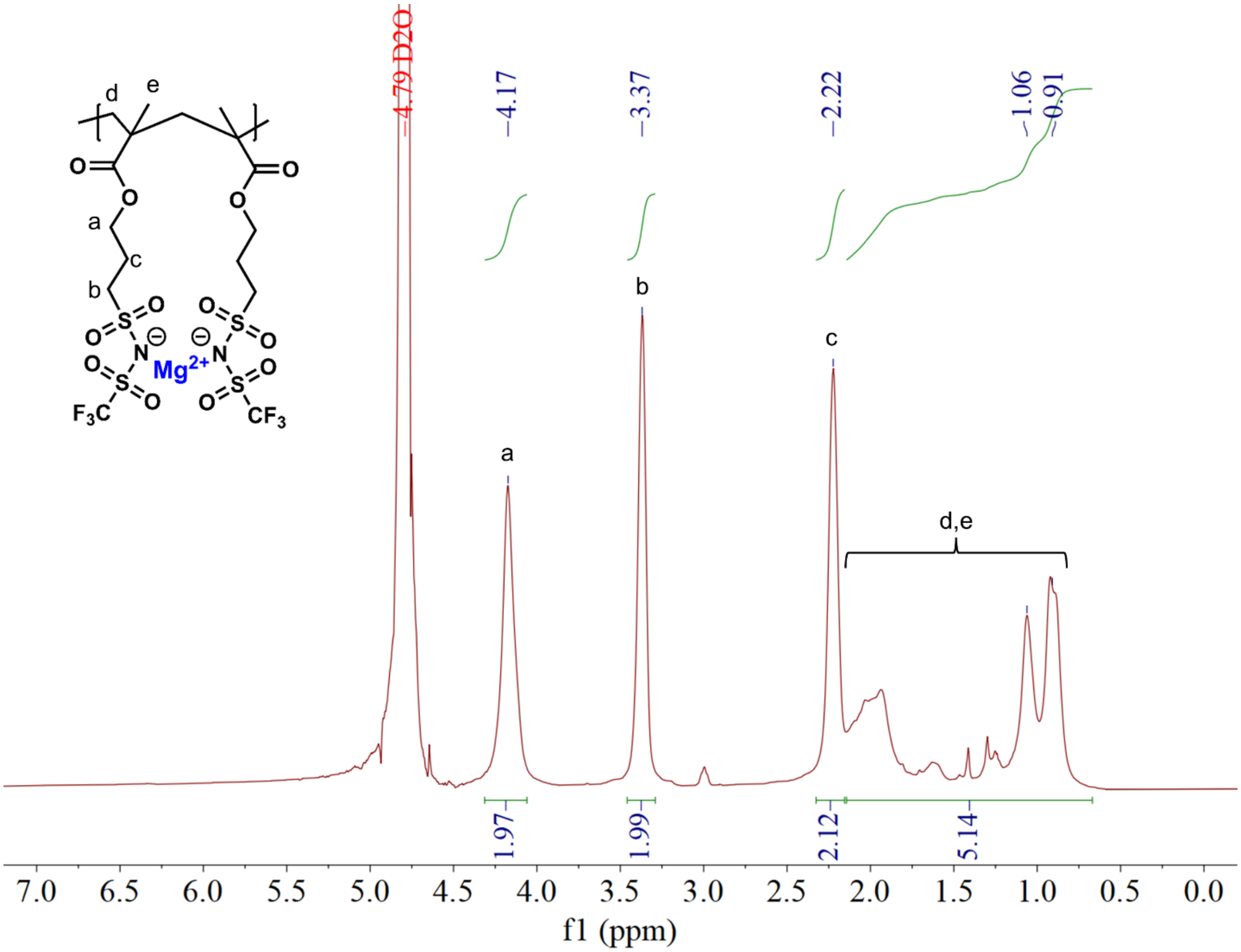

Figure S3. ^{1}H-NMR spectrum of P(Mg(MTFSI)$_2$) (400 MHz, D_2O): δ 4.17, 3.37, 2.22, 1.06, 0.91.

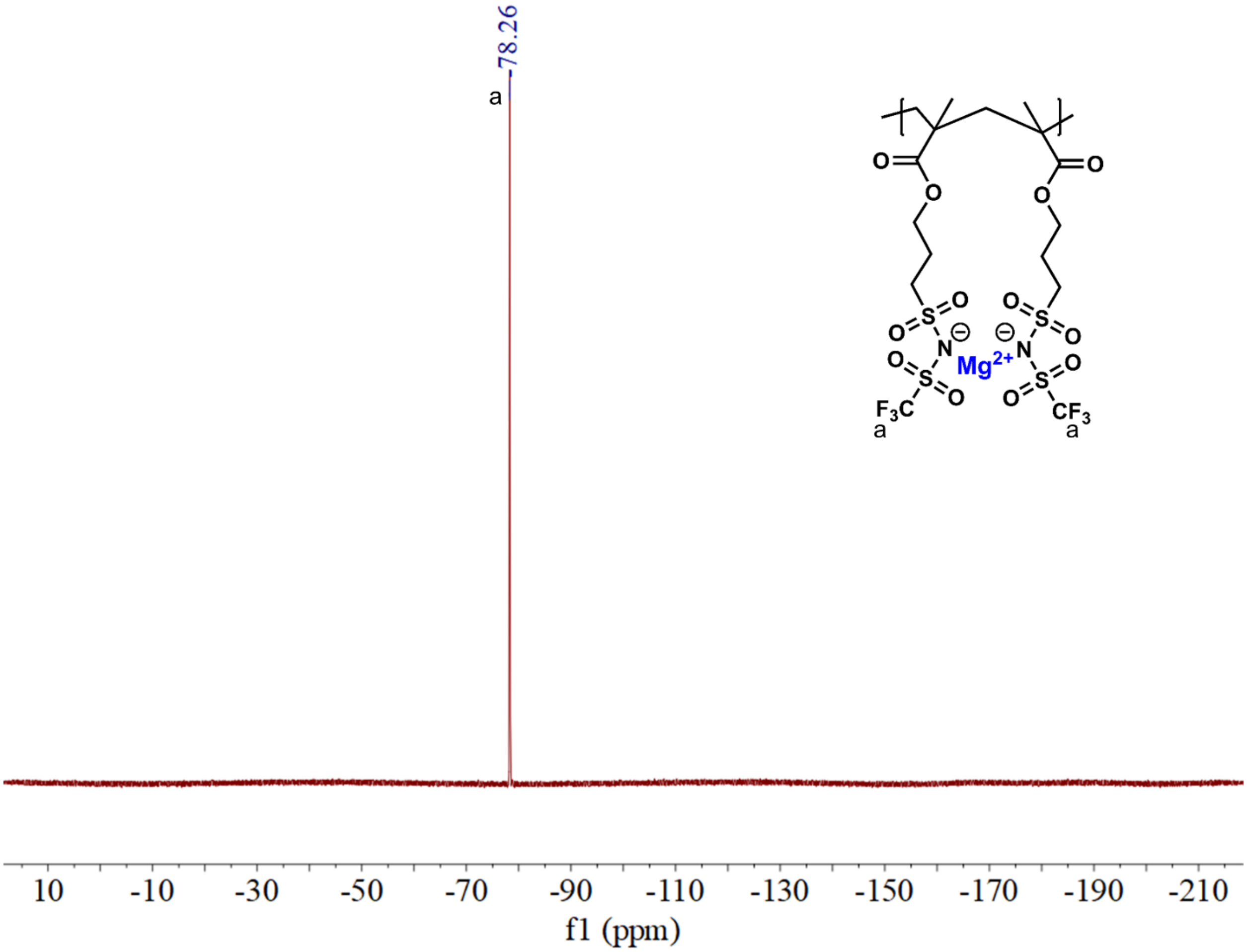

Figure S4. ^{19}F -NMR spectrum of P(Mg(MTFSI)$_2$) (377 MHz, D_2O): δ -78.26.

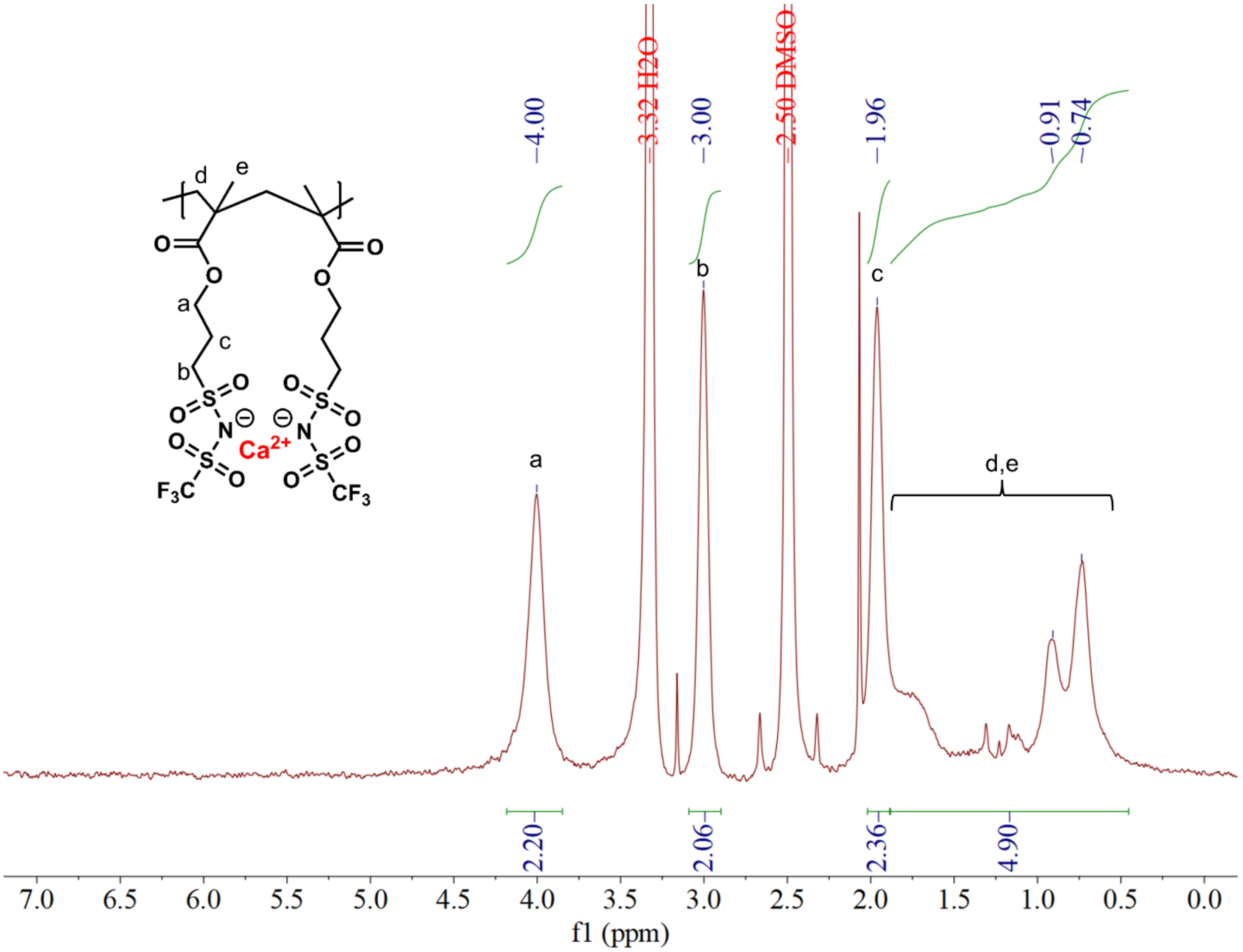

Figure S5. ^{1}H-NMR spectrum of P(Ca(MTFSI)$_2$) (400 MHz, D2O): δ 4.00, 3.00, 1.96, 0.91, 0.74.

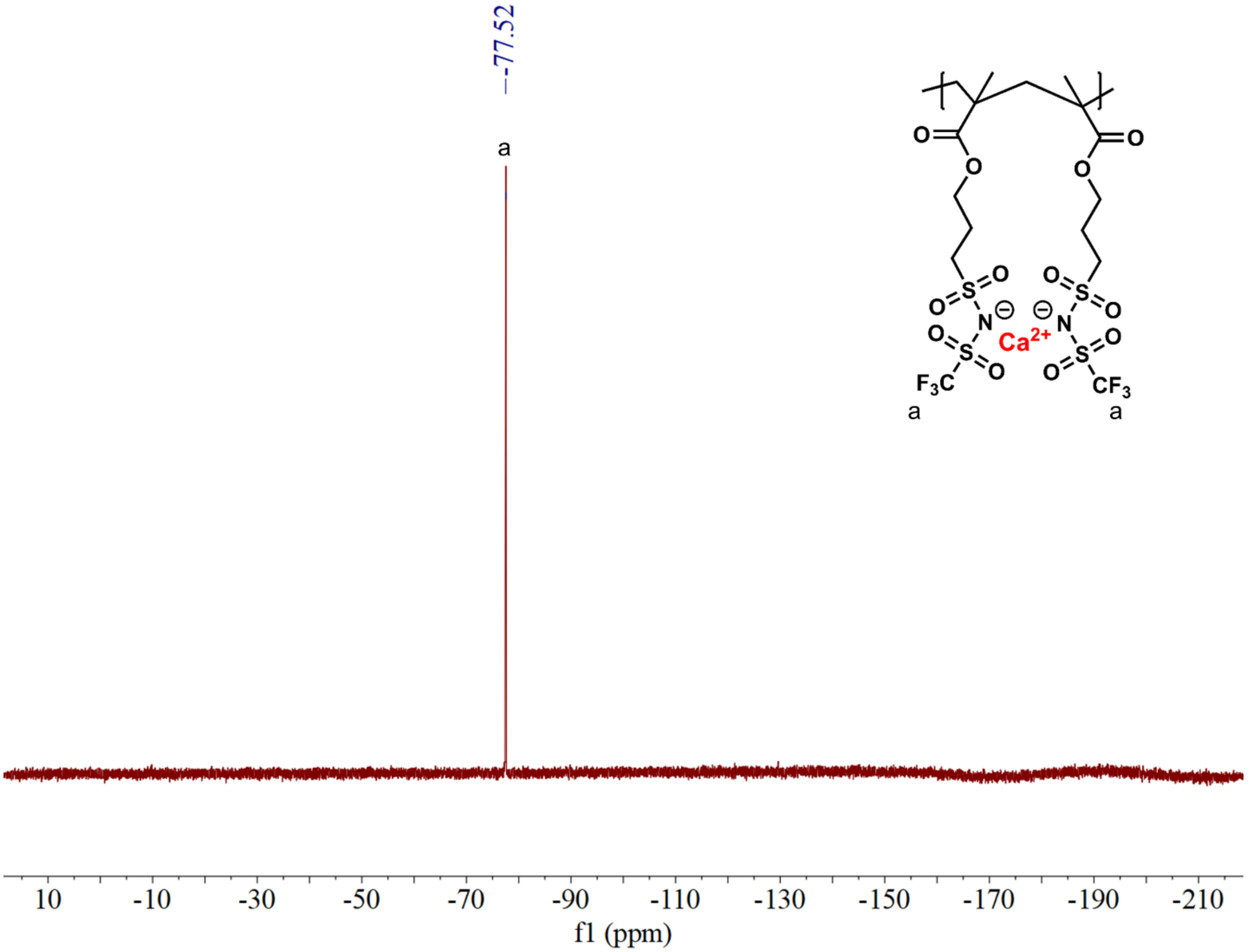

Figure S6. ^{19}F -NMR spectrum of P(Ca(MTFSI)$_2$) (377 MHz, D2O): δ -77.52.

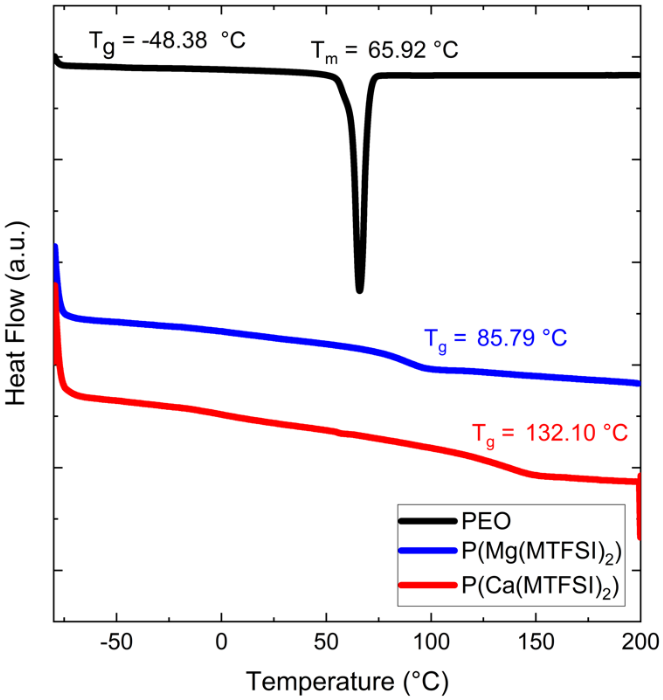

Figure S7. DSC thermograms of PEO, P(Mg(MTFSI)$_2$), and P(Ca(MTFSI)$_2$).

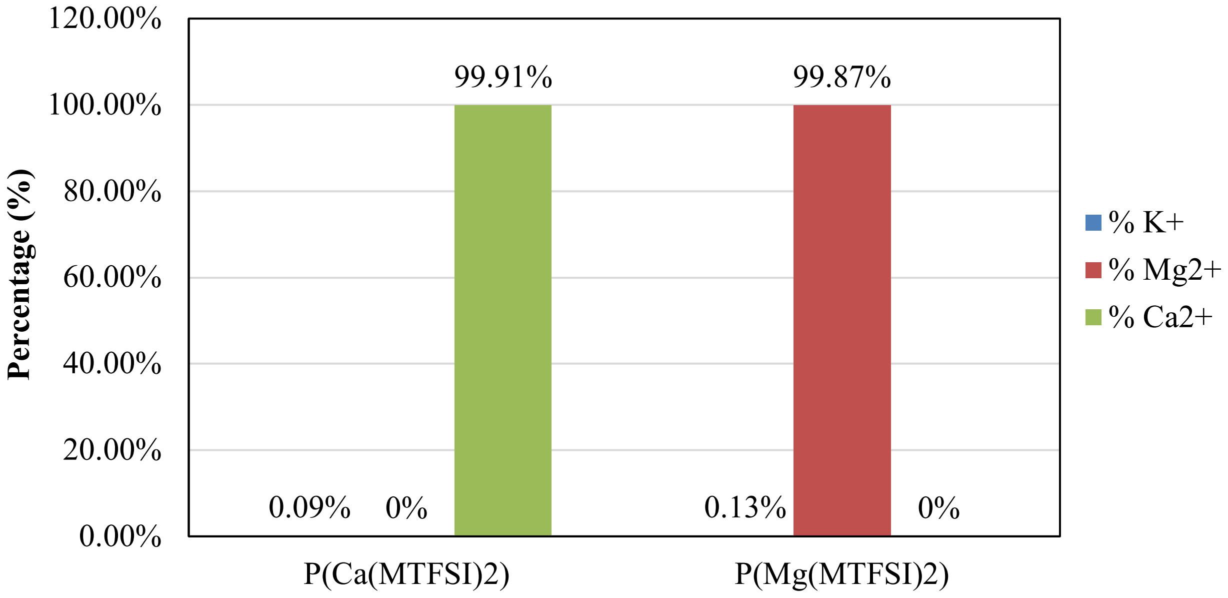

Figure S8. ICP-OES results of metal content in P(X(MTFSI)$_2$) (X = Mg^{2+}, Ca^{2+}).

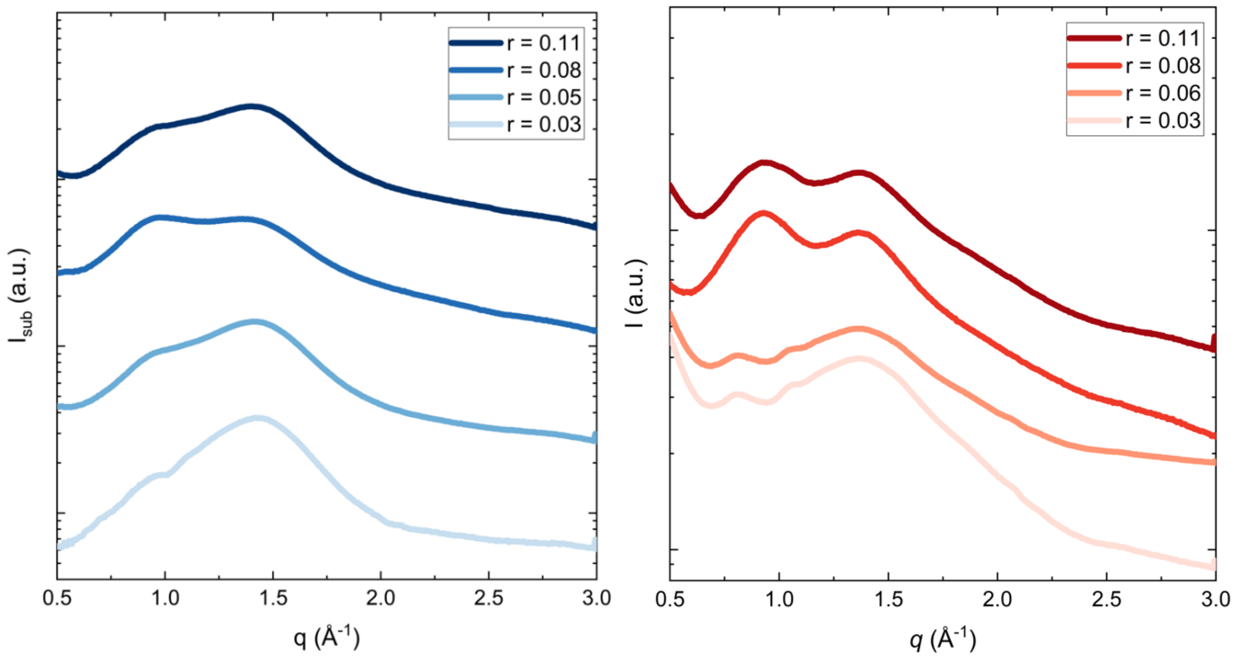

Figure S9. Wide-angle X-ray scattering (WAXS) profiles of PEO blends with (a) P(Mg(MTFSI)$_2$) and (b) P(Ca(MTFSI)$_2$) as a function of mixing ratio, r, at 90 °C. Error bars represent standard deviation and are smaller than the data points.

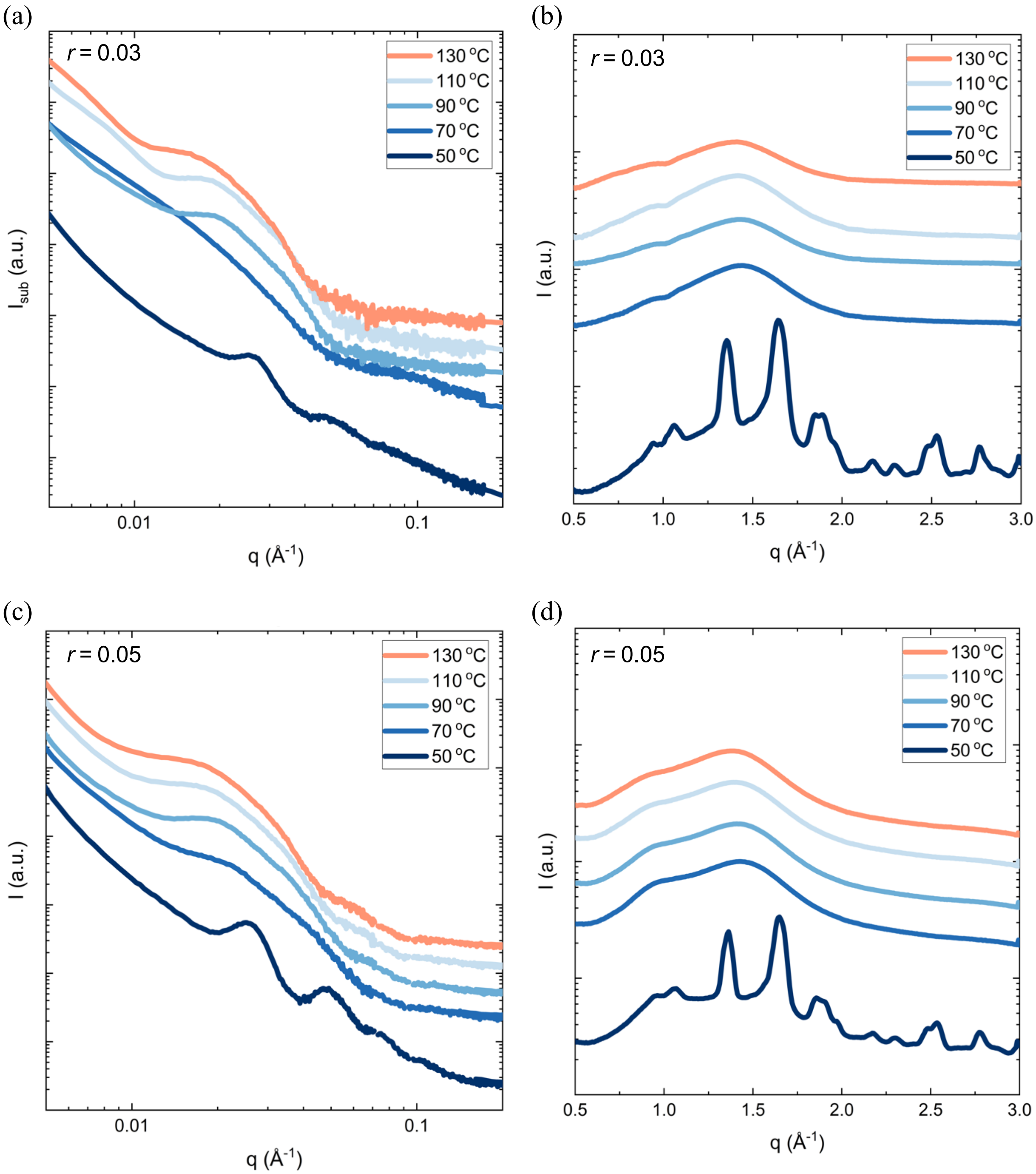
(a)
r = 0.03
130 °C
110 °C
90 °C
70 °C
50 °C
I sub (a.u.)
0.01
0.1
q (Å-1)
(b)
r = 0.03
I (a.u.)
0.5
1.0
1.5
2.0
2.5
3.0
(c)
r = 0.05
I (a.u.)
(d)
r = 0.05

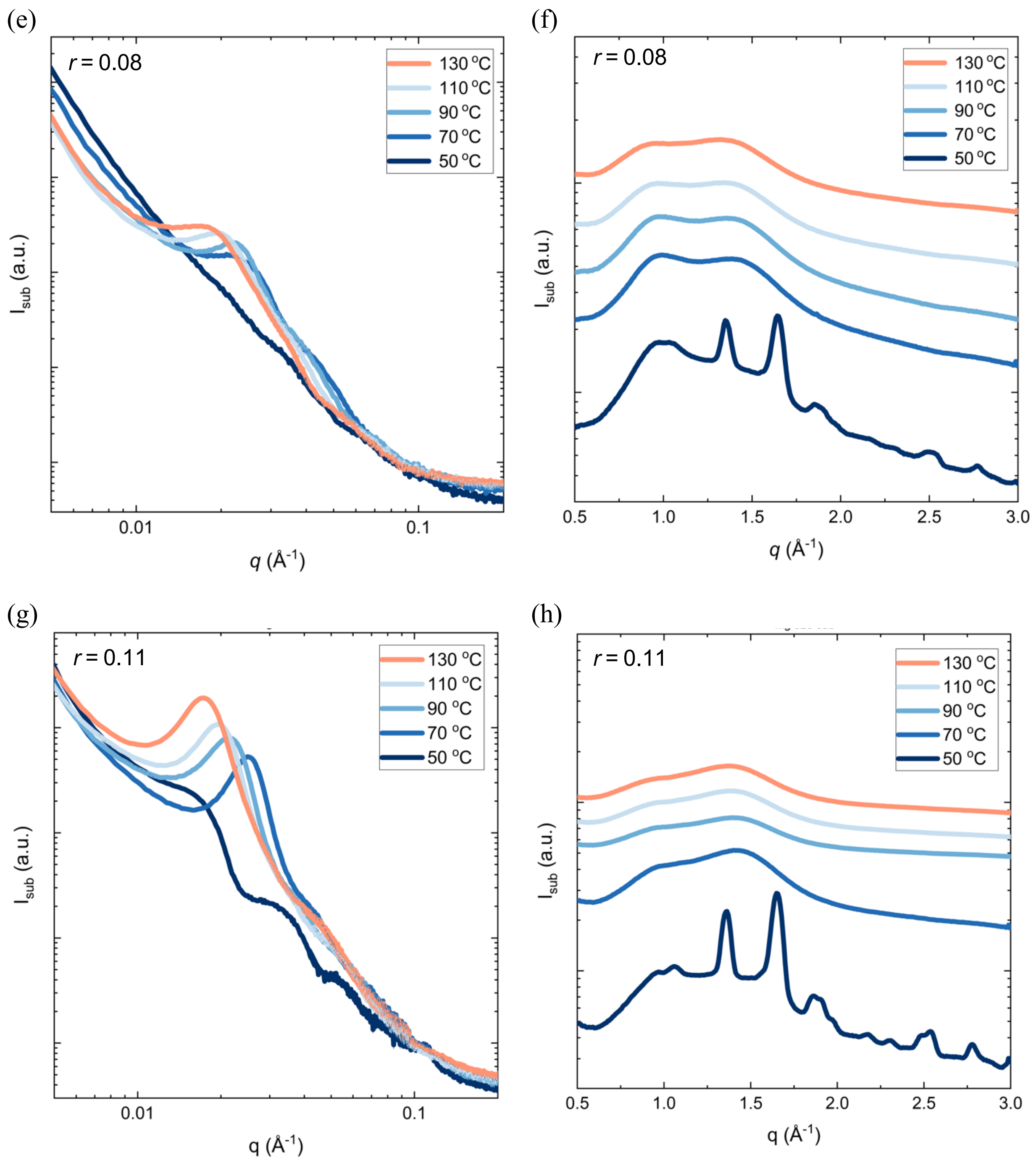

Figure S10. SAXS (odd-numbered) and WAXS (even-numbered) profiles of PEO/P(Mg(MTFSI)$_2$) blends recorded from 50 °C to 130 °C. Each pair of figures (a-b, c-d, e-f, g-h) corresponds to the same mixing ratio, r. Error bars represent standard deviation and are smaller than the data points.

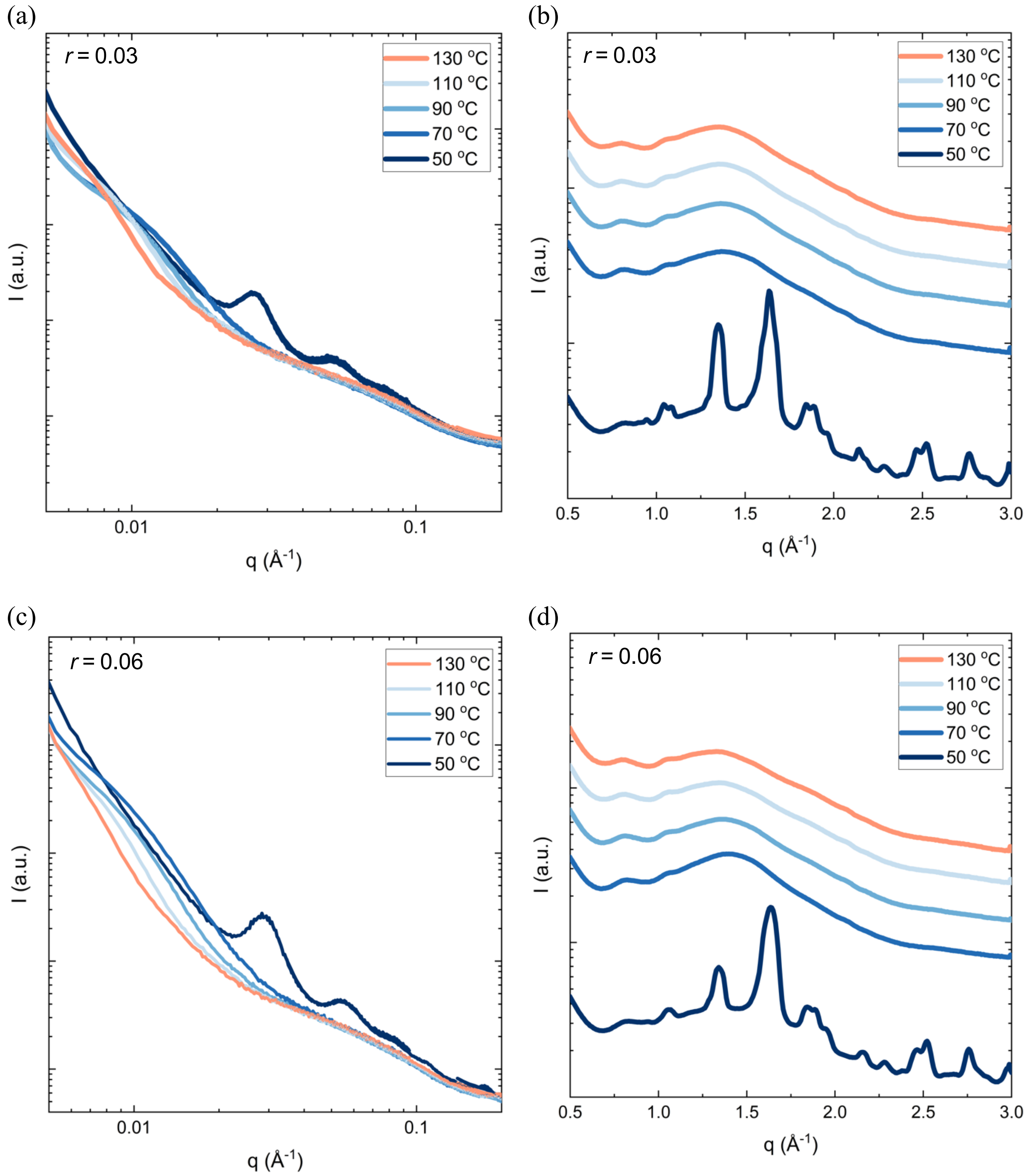

(a)
r = 0.03
130 °C
110 °C
90 °C
70 °C
50 °C
I (a.u.)
0.01
0.1
q (Å⁻¹)
(b)
r = 0.03
130 °C
110 °C
90 °C
70 °C
50 °C
I (a.u.)
0.5
1.0
1.5
2.0
2.5
3.0
q (Å⁻¹)
(c)
r = 0.06
130 °C
110 °C
90 °C
70 °C
50 °C
I (a.u.)
0.01
0.1
q (Å⁻¹)
(d)
r = 0.06
130 °C
110 °C
90 °C
70 °C
50 °C
I (a.u.)
0.5
1.0
1.5
2.0
2.5
3.0
q (Å⁻¹)

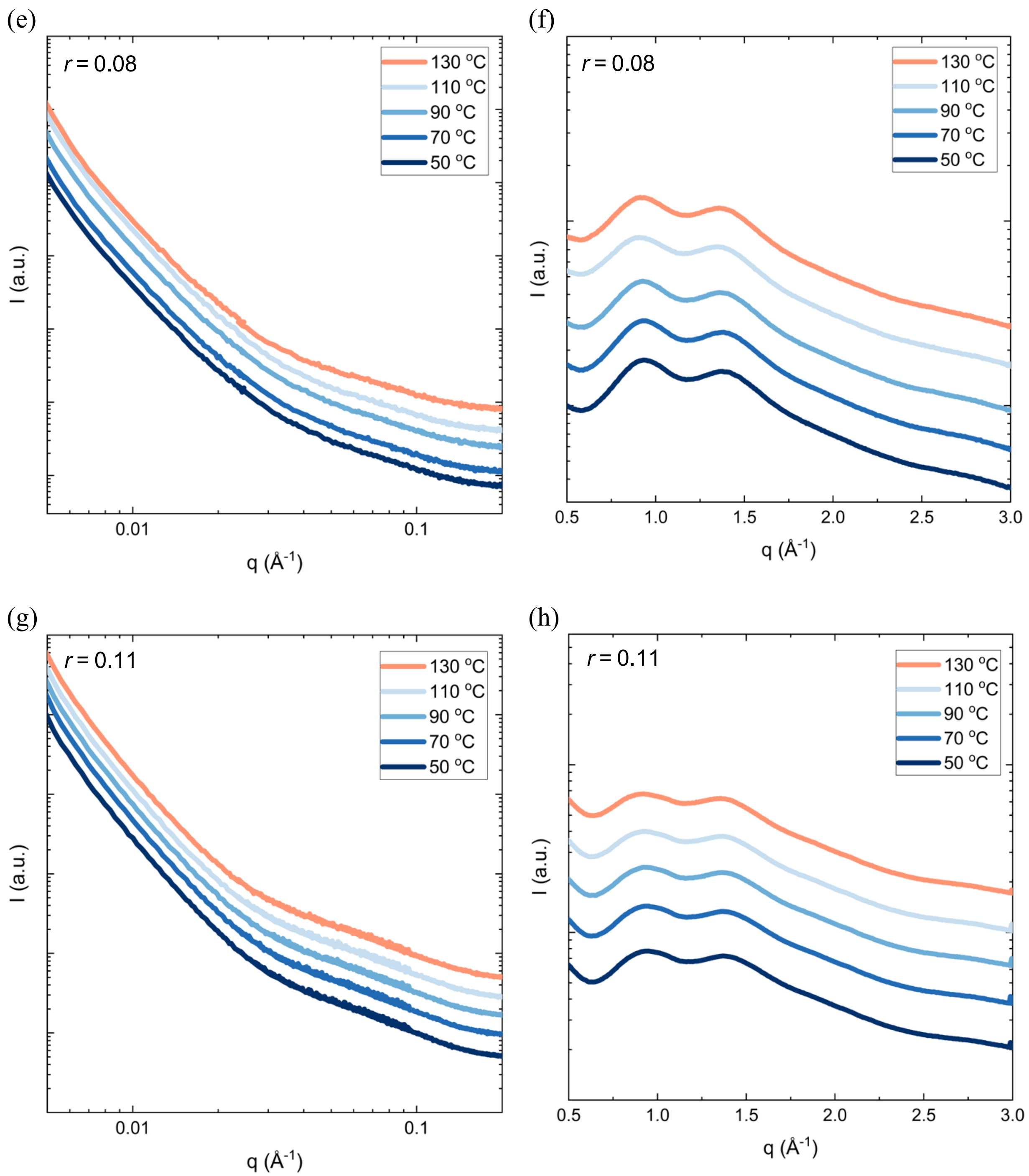

Figure S11. SAXS (odd-numbered) and WAXS (even-numbered) profiles of PEO/P(Ca(MTFSI)$_2$) blends collected from 50 °C to 130 °C. Each pair of figures (a-b, c-d, e-f, g-h) corresponds to the same mixing ratio, r. Data are presented as measured without subtraction of scattering from the Kapton tape. Error bars represent standard deviation and are smaller than the data points.

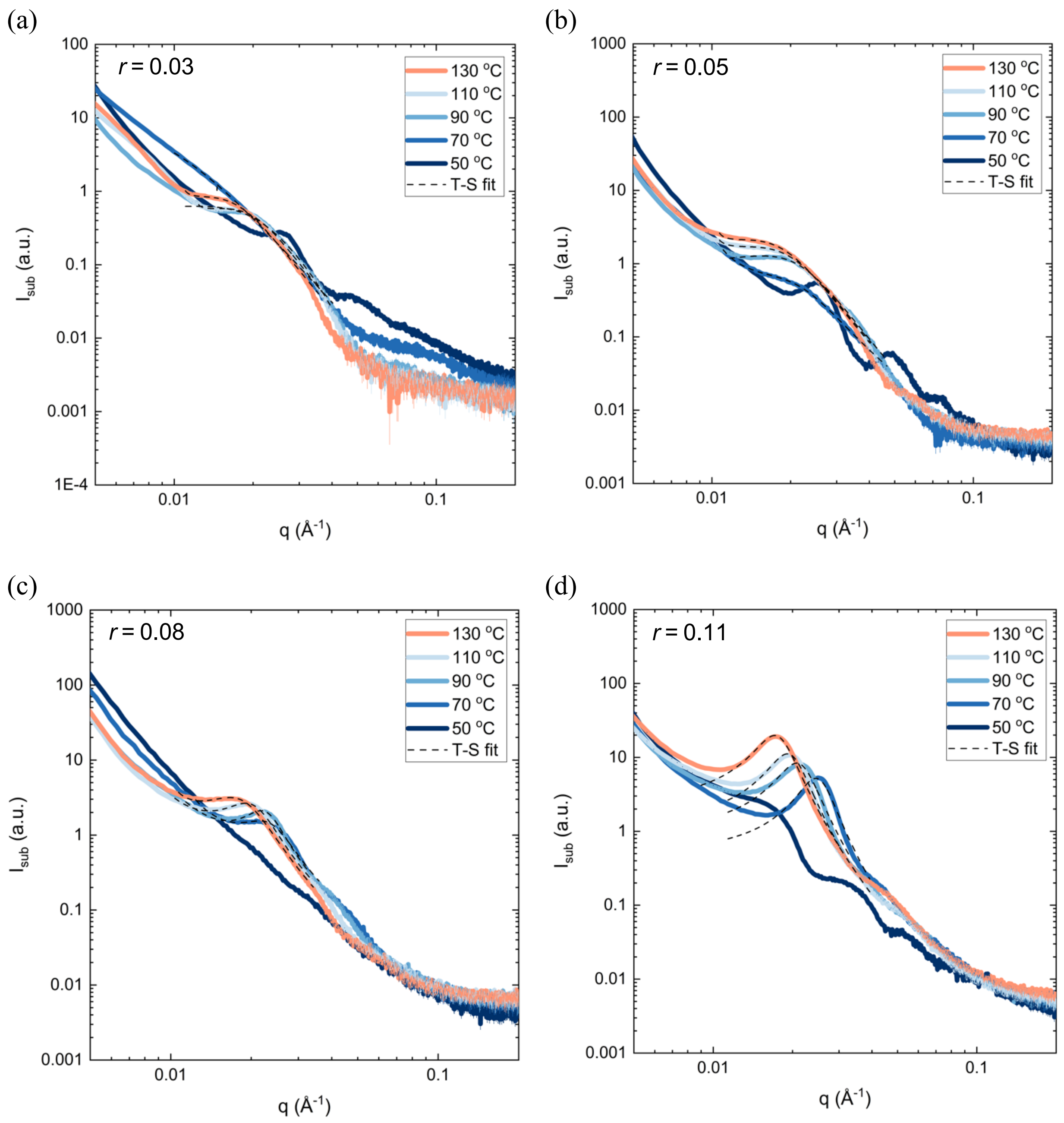

Figure S12. SAXS profiles of PEO/P(Mg(MTFSI)$_2$) blends collected from 50 °C to 130 °C for *r* = (a) 0.03, (b) 0.05, (c) 0.08, and (d) 0.11. Black dashed lines represent the Teubner-Strey (T-S) model that fits the data.

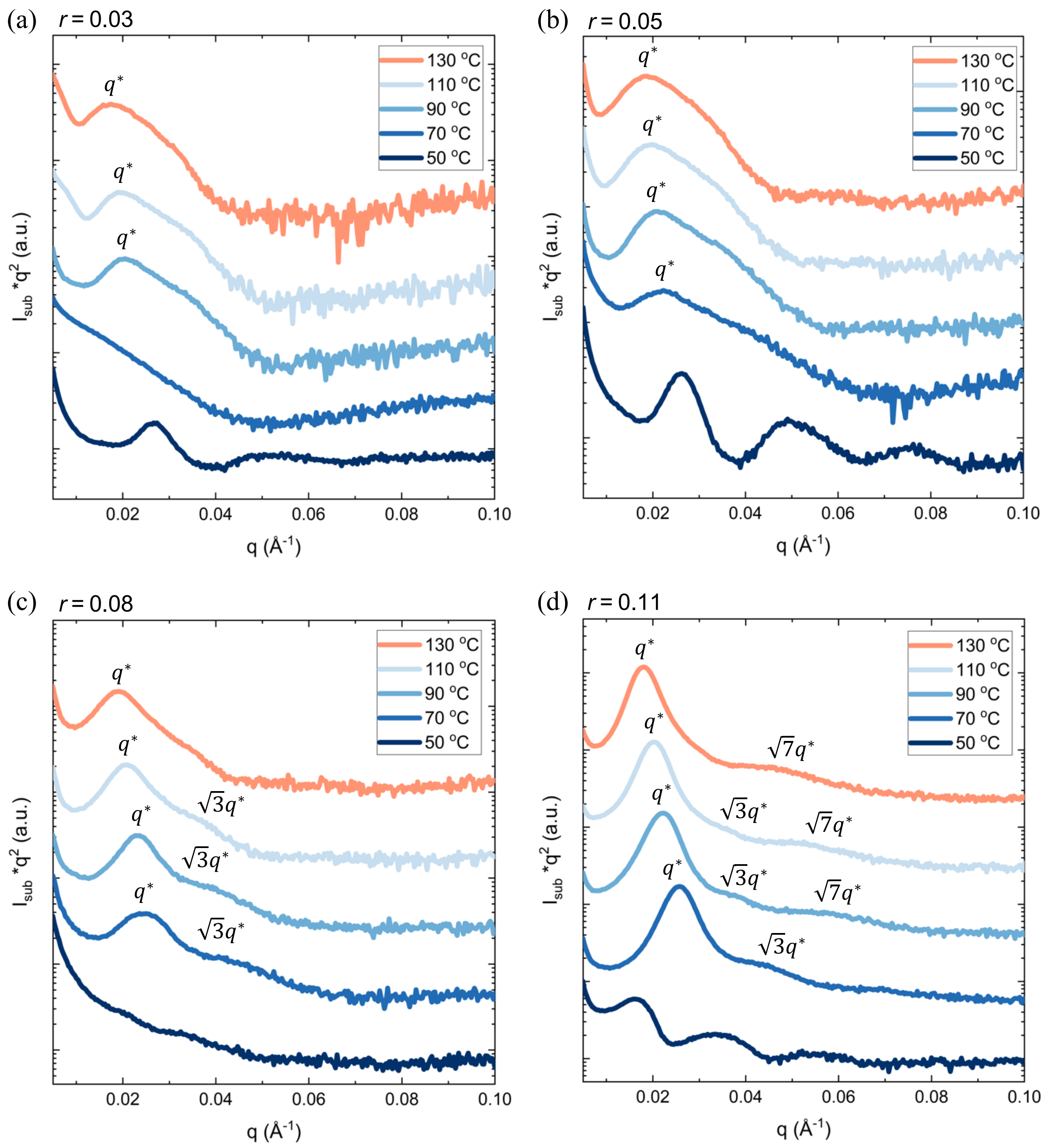

Figure S13. $I_{sub} \times q^2$ versus q profiles of PEO/P(Mg(MTFSI)$_2$) blends from 50 °C to 130 °C for r = (a) 0.03, (b) 0.05, (c) 0.08, and (d) 0.11.

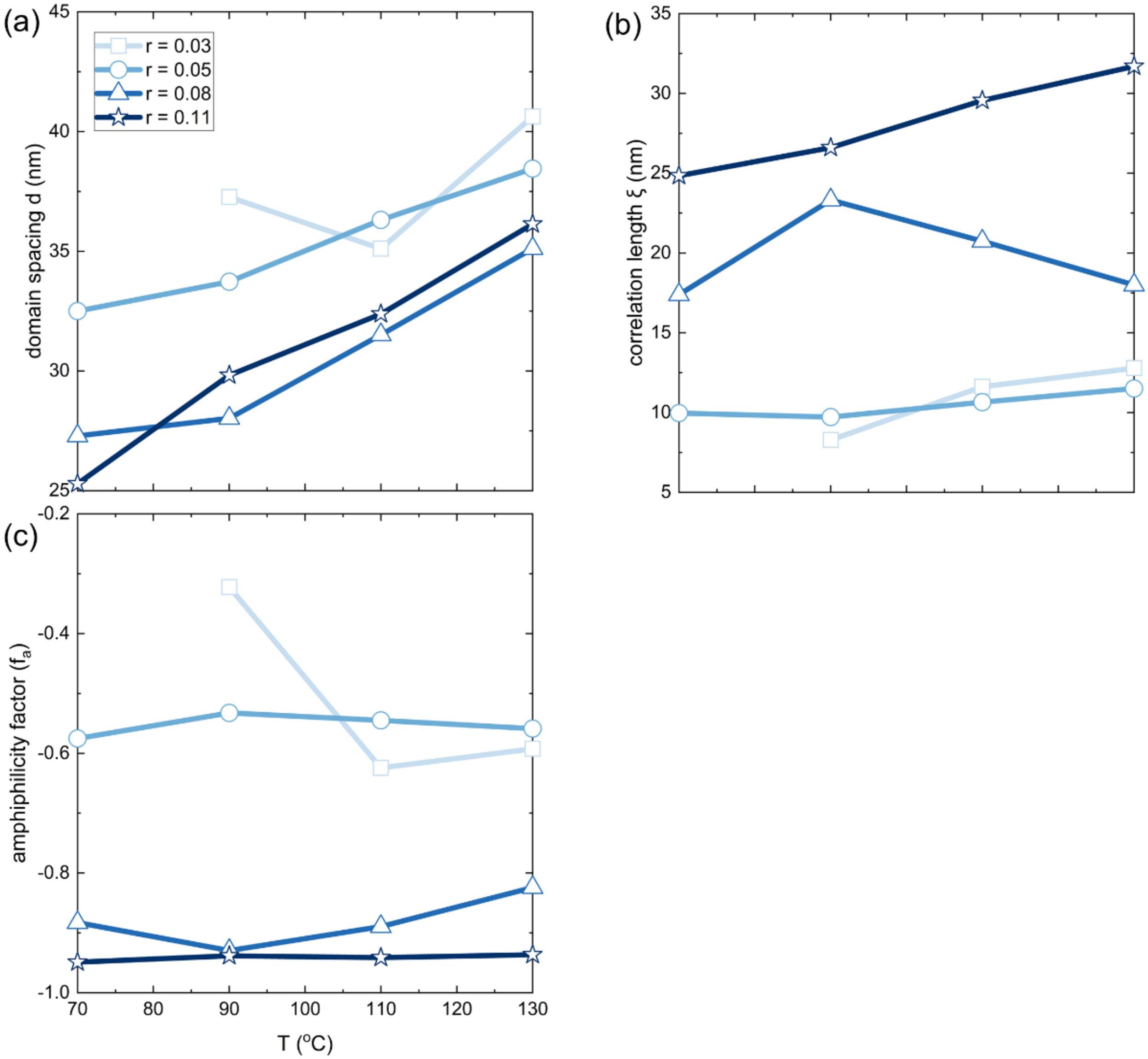

Figure S14. (a) Domain size, (b) correlation length, and (c) amphiphilicity factor of PEO/P(Mg(MTFSI)$_2$) obtained from fitting their scattering traces to the Teubner-Strey (T-S) equation.

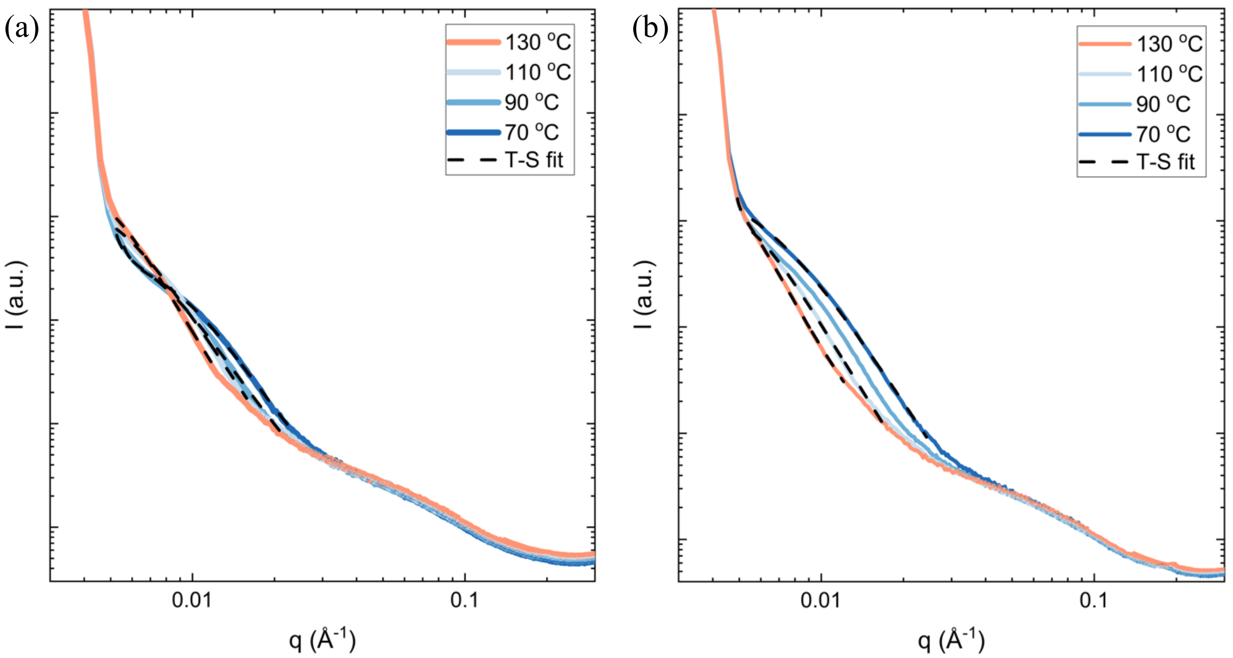

Figure S15. SAXS profiles of PEO/P(Ca(MTFSI)$_2$) blends collected from 70 °C to 130 °C for r = (a) 0.03 and (b) 0.05. Black dashed lines represent the Teubner-Strey (T-S) model that fits the data.

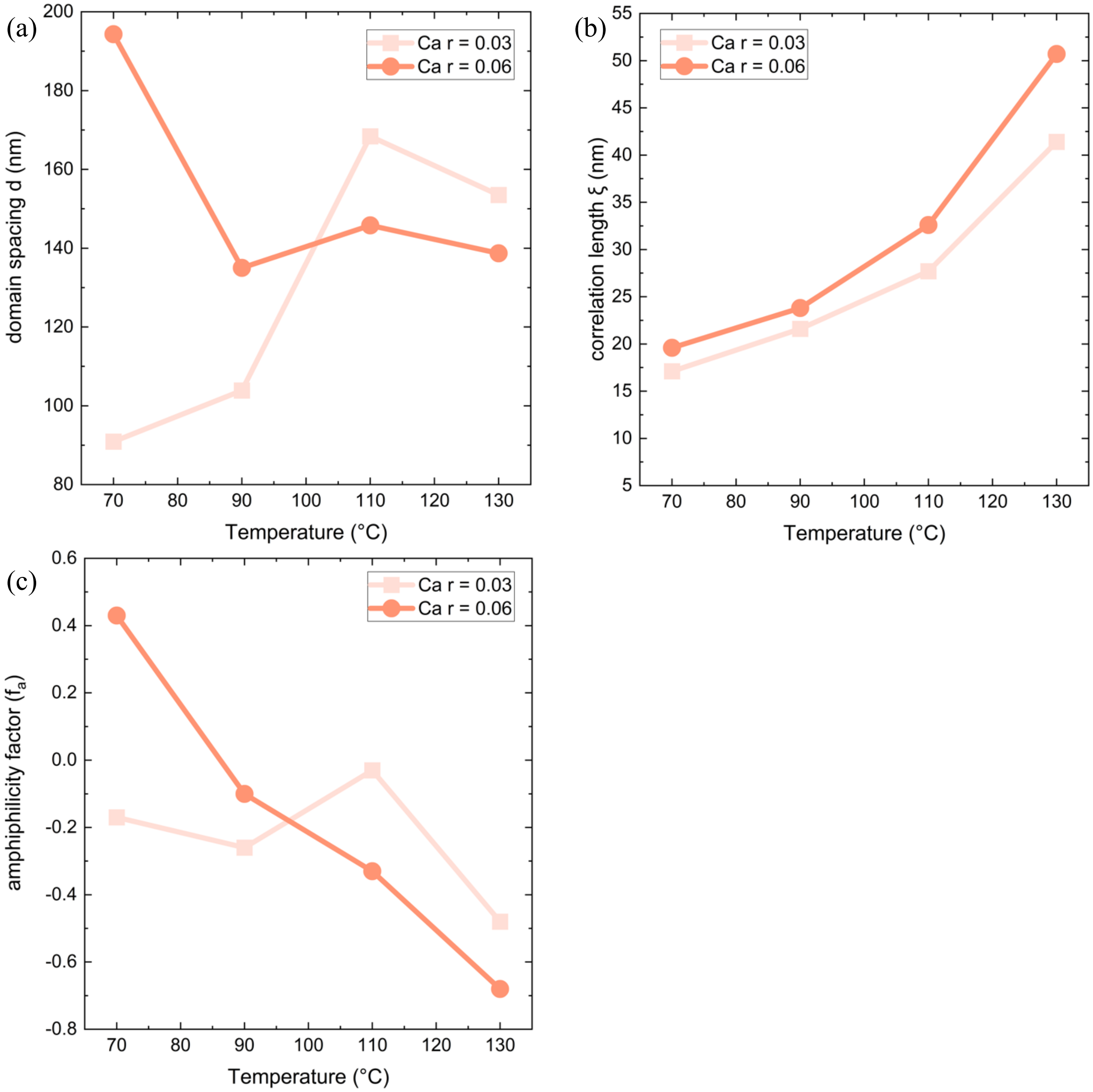

Figure S16. (a) Domain size, (b) correlation length, and (c) amphiphilicity factor of PEO/P(Mg(MTFSI)$_2$) obtained from fitting their scattering traces to the Teubner-Strey (T-S) equation.

References

(1) Wu, H.-J.; He, L.; Breining, W. M.; Lynn, D. M.; Loo, W. S. The Influence of Charge Correlation and Ion Solvation on the Phase Behavior of Single-Ion Conducting Polymer Blend Electrolytes Using SAXS/SANS. *Macromolecules* **2025**, *58* (16), 8866–8876. https://doi.org/10.1021/acs.macromol.5c00860.
